# Resistance of rocky intertidal communities to oceanic climate fluctuations

**DOI:** 10.1371/journal.pone.0297697

**Published:** 2024-05-29

**Authors:** Sarah A. Gravem, Brittany N. Poirson, Jonathan W. Robinson, Bruce A. Menge

**Affiliations:** Department of Integrative Biology, Partnership for Interdisciplinary Studies of Coastal Oceans (PISCO), Oregon State University, Corvallis, Oregon, United States of America; MARE – Marine and Environmental Sciences Centre, PORTUGAL

## Abstract

A powerful way to predict how ecological communities will respond to future climate change is to test how they have responded to the climate of the past. We used climate oscillations including the Pacific Decadal Oscillation (PDO), North Pacific Gyre Oscillation, and El Niño Southern Oscillation (ENSO) and variation in upwelling, air temperature, and sea temperatures to test the sensitivity of nearshore rocky intertidal communities to climate variability. Prior research shows that multiple ecological processes of key taxa (growth, recruitment, and physiology) were sensitive to environmental variation during this time frame. We also investigated the effect of the concurrent sea star wasting disease outbreak in 2013–2014. We surveyed nearly 150 taxa from 11 rocky intertidal sites in Oregon and northern California annually for up to 14-years (2006–2020) to test if community structure (i.e., the abundance of functional groups) and diversity were sensitive to past environmental variation. We found little to no evidence that these communities were sensitive to annual variation in any of the environmental measures, and that each metric was associated with < 8.6% of yearly variation in community structure. Only the years elapsed since the outbreak of sea star wasting disease had a substantial effect on community structure, but in the mid-zone only where spatially dominant mussels are a main prey of the keystone predator sea star, *Pisaster ochraceus*. We conclude that the established sensitivity of multiple ecological processes to annual fluctuations in climate has not yet scaled up to influence community structure. Hence, the rocky intertidal system along this coastline appears resistant to the range of oceanic climate fluctuations that occurred during the study. However, given ongoing intensification of climate change and increasing frequencies of extreme events, future responses to climate change seem likely.

## Introduction

Many species on Earth are sensitive to climate change [1–5]. However, the response of entire ecological communities and ecosystems to the changing climate is underexplored. Determining the responses of complex ecological communities comprised of a network of interacting primary producers, herbivores, predators, omnivores, and detritivores is typically untenable. However, some complex communities are easier to study than others. For example, rocky intertidal community members are relatively small, typically have slow-moving or sessile habits, mature and reproduce relatively quickly, and have a compact habitat that enables research at a community scale [6,7].

One approach for assessing community responses to global climate change is to amass long-term datasets of whole communities and explore relationships with past environmental variation [4,8]. Here, we report results of a 14-year study of responses of rocky intertidal communities in the northern California Current Large Marine Ecosystem (CCLME, coastal ecosystem from Baja California, Mexico, to British Columbia, Canada) to fluctuations in large-scale ocean climate and environmental patterns. These include the Pacific Decadal Oscillation (PDO), North Pacific Gyre Oscillation (NPGO), El Niño-Southern Oscillation (ENSO), as well as upwelling regime and variation (intermittency) in upwelling, maximum daily intertidal air temperatures, and average daily intertidal sea surface temperatures. We also tested the effects of the sea star wasting disease epidemic, which caused severe coast-wide declines in the keystone predator *Pisaster ochraceus* in 2013 and 2014 [9,10].

### Recent evidence of increasing instability

Our recent work shows some worrisome ‘early warning signals’ of impending change in this rocky intertidal system [11,12]. For example, annually repeated disturbance experiments to track recovery rates in the low zone at several sites in Oregon revealed increasing community instability (more variance in disturbed and undisturbed plots over time) and diminishing resilience (slower recovery from disturbance over time [11]). Further, overall declines and/or increasing variability in several key ecological metrics have occurred, including ecological subsidies to the nearshore from offshore (i.e. phytoplankton and invertebrate recruitment), mussel performance (body condition and reproductive output), and sea star performance (reproductive output and predation rates [12].

### Climate fluctuations may provide insight into drivers of community instability

Oscillating oceanic phenomena like the PDO (20–30 yr cycle), NPGO (7–15 yr cycle), and ENSO (3–7 year cycle), and environmental conditions like upwelling and air or sea temperatures have well-known impacts on some marine communities [13–18]. Higher PDO indices generally indicate warmer sea surface temperatures [13]. NPGO is orthogonal to PDO and higher indices generally indicate windier conditions and higher primary productivity [19,20]. In the CCLME, ENSO results in alteration between warmer, less productive El Niño years and cooler, more productive La Niña years [15,21,22]. Upwelling is caused by alongshore wind stress [23] that draws deep, nutrient-rich water up to the surface, and is usually strongest in June and July northern California and Oregon [24]. Variability in upwelling is also important, where intermittently strong upwelling (typical of central Oregon) provides both enough nutrients and enough wind relaxation for phytoplankton blooms to develop nearshore, while persistently strong upwelling winds (typical of southern Oregon and northern California) transports the blooms offshore [16,25]. Further, these environmental patterns and atmospheric climate are intricately linked, so global climate change is expected to alter them [17,26–29]. For example, the intensity and relative ecological dominance of NPGO versus PDO may be increasing [30,31], extreme El Niño and La Niña events are expected to increase [32]. On the other hand, expectations for upwelling are mixed [12,33,34]. Warming of both air and sea temperatures is ongoing and is expected to continue with climate change [12,35].

The oscillations in the metrics above provide a regime of past climactic variability that may help us understand marine community sensitivity or resistance to future climate change. For example, if communities were notably sensitive to environmental fluctuations over past decades, we may assume that communities will respond readily to climate change in the near future [36,37]. Conversely, if communities remain unchanged despite repeated climate fluctuations or perturbations, we may assume that these communities are more resistant [38]. While this line of inquiry may provide some insight for the near future, we admittedly do not know whether the relationships between increasing climate stress and community structure are linear. For example, communities may exhibit tipping points [39–41] where even gradual environmental change can lead to sudden community responses. Also, climate-related, prominent drivers of community change such as extreme events like heat waves, disease outbreaks, and natural disasters are clearly increasing in frequency [42–45]. Though predicting the effects of such episodic events is challenging, we may be able to infer whether communities will be responsive to future climate changes by testing their sensitivity to past variation in their environment. These include responses to extreme events in the CCLME, like the exceptionally strong El Niño event in 2015–2016 [46] and the marine heat wave dubbed ‘the blob’ that hit the Northeast Pacific in 2014–2015 [47].

### Sensitivity of processes and communities to oceanic climate variation

Along the West Coast of North America, spatial differences in average oceanic conditions are known to be important drivers of intertidal community structure [25,48–53]. For example, community structure clearly varies along this coastline, and this has been attributed to several environmental drivers including temperature (air and sea), nutrient concentrations, upwelling, upwelling variation, precipitation, and tidal range [48,50,53]. Clearly, the *average* oceanic climate can have a strong influence on intertidal communities in different places, but here we focus on whether interannual *fluctuations* in oceanic conditions relate to community structure at a given location over time. Overall, we are interested whether intertidal communities are sensitive to interannual oceanic climate fluctuations, or instead remain consistent despite such changes.

Recent evidence suggests that multiple ecological processes at population and organismal levels are sensitive to oceanic variation along the Oregon California coasts. For example, monthly mussel recruitment tended to vary with NPGO and upwelling but not PDO or ENSO [36,54]. Barnacle recruitment also appears to be sensitive to upwelling [34,55,56], but not in all cases [57–59]. However, order-of-magnitude increases in mussel recruitment associated with changing climate regime in the early 2000s [20] did not translate into changes in community structure [20,36]. Further, while mussel recruitment was sensitive to environmental conditions, mussel cover was not, suggesting a decoupling between recruitment and community structure dynamics [60].

Oceanic climate can also affect growth rates and physiology of intertidal species. Higher phytoplankton abundance and warmer water during warm-phase PDO and El Niño years correlated with increased growth rates of mussels [61,62]. The kelp *Egregia menziesii* grew faster in a strongly upwelled region north of Point Conception, California than it did in the weakly upwelled region south of Point Conception [63]. Changes in seasonal and yearly upwelling, nutrients, water temperature, ENSO and PDO correlated to changes in growth rates and Carbon:Nitrogen ratios in multiple dominant macrophyte species along the Oregon Coast [64,65]. The studies above also highlight how studies lasting just several years to one or two decades can successfully detect the ecological consequences of climate oscillations operating on multi-decadal scales (e.g. PDO, NPGO).

Increasing evidence suggests that rocky intertidal community structure is already responding to climate change. Smith et al. [66] documented lower diversity in California rocky intertidal communities in 2002 compared to the 1960s and 1970s and suggested either a warm phase of PDO or warming global temperatures were responsible. Poleward range shifts of rocky shore species has occurred in northern Europe [67], and the relative dominance of northern versus southern species has shifted in California [68]. Globally, many coastal ecosystems have shown sometimes dramatic susceptibility to warming climate [e.g. 69–72]. Here, we examine if annual shifts in intertidal community structure in Oregon and northern California correlate to oceanic environmental variation to identify which, if any, environmental factors may drive community change. By identifying these drivers, we may be better equipped to predict and perhaps mitigate the effects of changing climate on these iconic communities.

### Approach

Apart from the simple hypotheses that rocky shore communities did not (null) or did (alternative) respond to recent changes in ocean climate, our approach was primarily focused on discovering patterns. To determine if intertidal community structure changed during a period of intensifying climate change [35], we used a 14-year dataset (2006–2020) taken at 11 sites in Oregon and northern California spanning 294 km (183 miles) of coastline. We investigated whether these communities were sensitive to yearly variation in PDO, NPGO, El Niño, upwelling intensity, upwelling variability, air temperature and water temperature during this time. We also included a time series of sea star wasting disease occurrence in our analysis because it caused coast-wide declines in the abundance of the keystone predator, the sea star *Pisaster ochraceus* [9], so may have caused strong ecological effects in this system [73,74]. Though wasting disease was more severe in warmer locations [75], there is either no or modest evidence that the outbreak was triggered by environmental factors [9,10,76–78]. Because the periodicity of some climate oscillations (e.g., PDO, NPGO) is typically longer (10–30 yr) than most ecological data sets, our ability to investigate the full extent of their importance is limited. However, when multidecadal datasets are unavailable, examining the relationships between ecological patterns and yearly variation in these climate indices over several years can provide insight into their potential importance [20,54,62]. We quantified percent cover of sessile taxa and density of mobile taxa yearly in the high, mid and low intertidal. We asked 1) how have intertidal community structure, abundance of focal functional groups, and diversity varied over time? We then investigated sensitivity of these communities to climate variation by asking 2) have the abundance of focal functional groups, rate of community change, or diversity correlated with yearly fluctuations in PDO, NPGO, El Niño, upwelling intensity, upwelling variability, air and water temperatures, and sea star wasting disease?

## Materials and methods

### Community surveys

**Fig 1:**
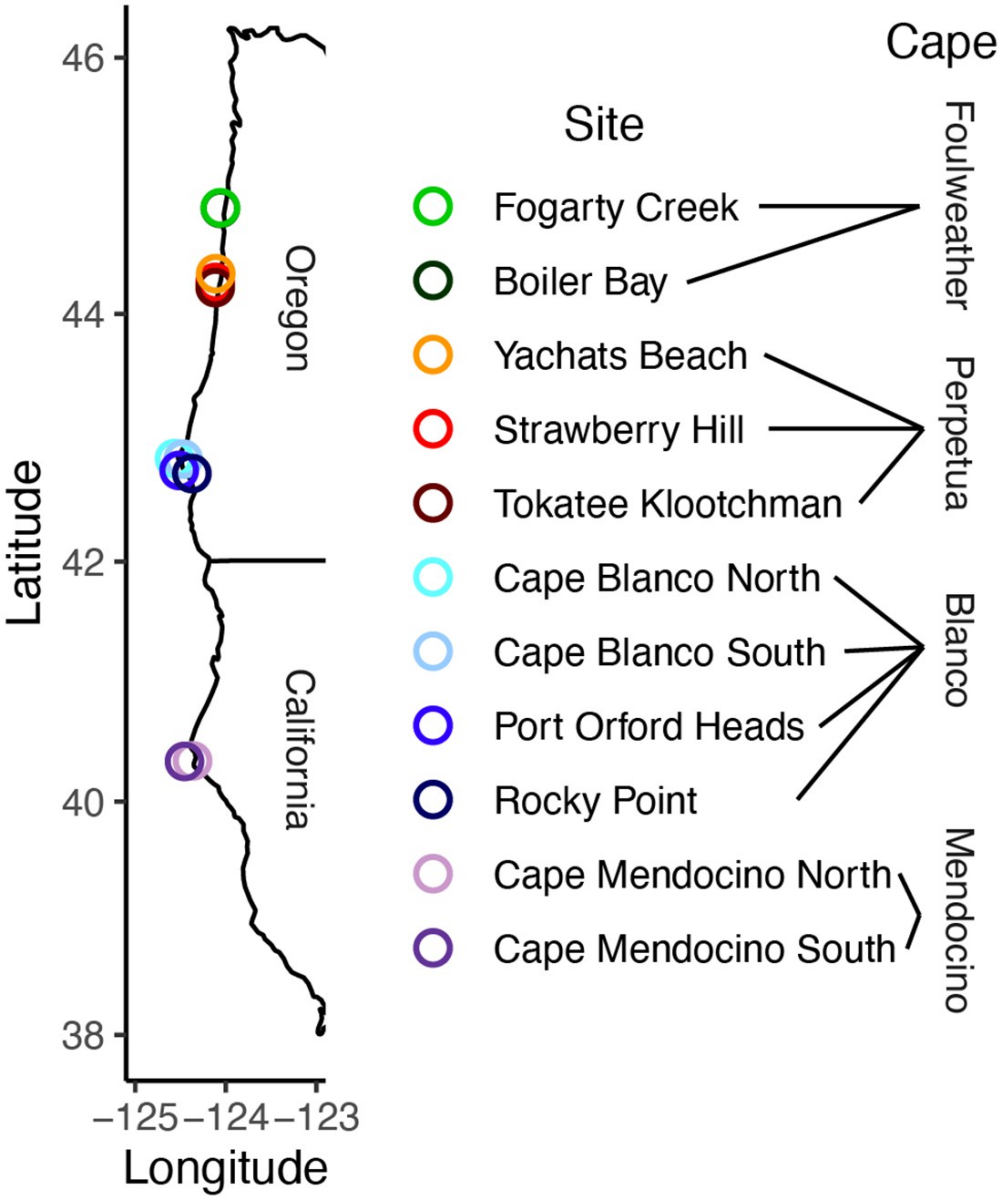
Map of sites in Oregon and northern California. Capes included Cape Foulweather in greens (Boiler Bay and Fogarty Creek sites), Cape Perpetua in reds (Yachats Beach, Strawberry Hill and Tokatee Klootchman sites), Cape Blanco in blues (Cape Blanco North, Cape Blanco South, Port Orford Heads and Rocky Point sites), and Cape Mendocino in purples (Cape Mendocino North and South sites). See S1 Table in S1 Appendix for coordinates.

Starting in 2006, summer (June-August) community surveys were conducted annually at 11 sites on 4 capes (i.e., headlands or regions) from central Oregon to northern California (Fig 1, S1 Table in S1 Appendix). From north to south, Capes included Cape Foulweather (sites Fogarty Creek and Boiler Bay), Cape Perpetua (sites Yachats Beach, Strawberry Hill and Tokatee Klootchman), Cape Blanco (sites Cape Blanco North and South, Port Orford Heads and Rocky Point), and Cape Mendocino (sites Cape Mendocino North and South). Scientific collection permits for this work were granted by the Oregon Department of Fish and Wildlife (ODFW 19272, 21042, 21848) and the California Department of Fish and Wildlife (SC-4055, S-182930001-18295-001). With exceptions noted below, we surveyed communities separately in each of 3 intertidal zones, with the low zone defined as below mussel beds, the mid zone as mussel beds, and the high zone as above mussel beds. Because of time constraints or lack of habitat, we performed only low zone surveys but not mid or high surveys at Tokatee Klootchman, Cape Blanco South, Port Orford Heads and both Cape Mendocino sites. In addition, we began most mid and high zone surveys in 2011, five years after the low zone surveys. Because the low zone is patchier and more diverse than the mid and high zones, we used three 30 m transect line(s) parallel to the shore in the low zone at least 20 m apart versus one 30 m transect line in mid and high zones. On each transect, we uniformly (every three meters along transect tapes in mid and high zones) or haphazardly (quadrat tosses in the low zone, avoiding sand, tide pools and large crevices) sampled 10 0.25m^2^ quadrats (total of 4,588 quadrats). Because we sampled the low zone more thoroughly than the mid and high zones, this may affect metrics like biodiversity and community structure at the site level, so we analyzed zones separately in all except one model in this study (See Data Analyses). We performed surveys in the same general area each year, but the quadrat locations varied between years. In each gridded quadrat, we visually estimated the percent cover to the nearest 1% of all visible species, including sessile algae, sessile invertebrates and bare space and counted total numbers of all mobile macroinvertebrates. Due to layering of macrophytes or the epibionts growing on mussels, cover could total >100% when summing all species surveyed. We identified biota to species whenever possible, but those that we were not able to identify reliably to species or were very rare were lumped at the genus level or higher for diversity metrics (see S2 Table in S1 Appendix for species list).

### Oceanic climate variables

Climate indices used here oscillate on a 20–30 year scale for the Pacific Decadal Oscillation (PDO), 7–15 years for the North Pacific Gyre Oscillation (NPGO), 3–7 years for El Niño Southern Oscillation (ENSO), and inter- and intra-annually for upwelling. Please see S2 Appendix for a brief background on these oceanic climate variables and how each may be affected by global climate change. We downloaded data for each of these oscillations for the northern California Current System between 2006 and 2020, including the PDO from the National Centers for Environmental Information at NOAA [13,79,80], the NPGO index from the NPGO portal [19], the ENSO from NOAAs Physical Sciences laboratory using Multivariate ENSO Index Version 2 (MEI.v2) [22,81], and upwelling as the monthly means of daily indices of the Biologically Effective Upwelling Transport Index BEUTI at 1° resolution, [82,83]. PDO, NPGO and ENSO are ocean basin-wide measurements [13,19,22], so the values were the same for all sites each year. For upwelling, we used the latitude appropriate for each cape (44°N for Capes Foulweather and Perpetua, 42°N for Cape Blanco and 40°N for Cape Mendocino). For ENSO indices, we assigned the bimonthly designation (e.g. DecJan) to the latter month (e.g. January, [22]. We measured intertidal sea surface and air temperatures every 15 minutes at each site using temperature-logging sensors (HOBO Tidbits or HOBO Pendants, Onset Corp., Bourne, MA). We separated air from water temperatures using a de-tiding method coupled with tide tables (http://tidesandcurrents.noaa.gov) [62]. We then calculated the maximum daily air temperature and the mean daily water temperature at each site. For sea star wasting disease, we included the years before or since the SSWD outbreak at each site, with zero, negative, and positive years demarcating the onset of, prior to, and after SSWD in 2013 at California sites and 2014 at Oregon sites [9,84]. Since some of the indices have a seasonal component during which their effects are most prominent, we first determined the window of peak months as from Oct—Mar for PDO, Dec—Mar for NPGO, Dec—Apr for ENSO, March to the summertime survey month for upwelling, and April to the survey month for maximum air temperature [22,85–87]. We calculated the averages among months for each index during those peak months only, and then assigned this average to the subsequent annual community survey. Since upwelling intermittency can have strong effects on community processes [16,25], we estimated this metric using the standard deviation of the mean upwelling index from March to the survey month.

### Data analyses

Our overarching goal was to investigate if intertidal community structure varied over time and space, then test whether temporal variation was associated with interannual oceanic climate variation. We performed all analyses in Primer-e 7 with PERMANOVA+ add-on [88,89], JMP Pro v15 (SAS Institute), R v4.0.0 [90] and R Studio v1.2.5042 (R Studio Inc.). Below, we summarize our general approach for community analyses, then specific features of each model.

#### Multivariate community structure analyses

*General approach*. For all statistical models, we first aggregated the species data to the functional group level (see S2 Table in S1 Appendix for list) then log_10_-transformed abundances to increase or decrease the influence of rare or common taxa, respectively. We quantified community similarity using Bray-Curtis resemblance matrices. We tested the relative importance of temporal (year), spatial (e.g. zone, site, cape), or oceanic climate (climate metrics and years since SSWD) factors on community structure using multiple distance-based permutational analyses of variance (PERMANOVA) [91,92]. We used 999 permutations under a reduced model, conditional type 2 sums of squares, and employed multivariate pair-wise comparisons among levels of significant factors. Percent contribution of each model term to the overall fit was determined by dividing the term’s component of variation to total model variation [89]. We used similarity percentages analysis (SIMPER) to identify the functional groups substantially contributing to dissimilarity among or similarity within levels of a factor [88]. We visualized community separation for each term using non-metric multidimensional scaling (nMDS) plots with vector overlays for those functional groups having the strongest correlations to nMDS cloud separation. We used PERMDISP to determine if each significant term in PERMANOVAs met the assumptions of multivariate homogeneity [89,93] (see SI for details).

*Community structure among zones*. Zone effects on community structure were analyzed by testing main and interacting effects of intertidal zones (high, mid or low), capes, sites (nested within capes) and years, with cape, site, and year as random factors using PERMANOVA. Since the dataset was large and thus computer processing was prohibitively time-consuming, we averaged each functional group by transect rather than including each quadrat separately. Unsurprisingly, community structure was overwhelmingly driven by intertidal zone (Table 1, see Results). Since our primary focus was on temporal variation, we performed further analyses separately by zone.

**Table 1:** Results of a) a PERMANOVA analysis testing the effects of zones, capes, sites, years, and their interactions on intertidal community dissimilarity in Oregon and Northern California from 2006–2021. Cape, site, and year were treated as random factors. b) Results of SIMPER analysis testing the contributions of taxa to within-zone similarity. Only the top ten taxa contributing most strongly to within-zone similarity are listed. Communities were analyzed at the transect level (the average of ~10 0.5 x 0.5m quadrats).

a) PERMANOVA for Zone, Cape, Site, and Year								
**Source**	**df**	**SS**	**MS**	**Pseudo-F**	**P**	**Estimate**	**Sq.root**	**% Explained**
**Zone**	2	281,830	140,920	18.43	0.001	1698.00	41.21	55.3
**Cape**	3	88,568	29,523	3.01	0.001	187.65	13.70	6.1
**Year**	14	53,610	3,829	4.10	0.001	97.61	9.88	3.2
**Site[Cape]**	7	53,955	7,708	11.77	0.001	190.69	13.81	6.2
Zone*Cape	4	24,899	6,225	2.28	0.001	146.05	12.09	4.8
Zone*Year	20	22,113	1,106	2.76	0.001	100.37	10.02	3.3
Cape*Year	38	31,580	831	1.26	0.008	23.00	4.80	0.7
Zone*Site[Cape]	6	14,146	2,358	6.31	0.001	164.28	12.82	5.3
Site[Cape]*Year	76	48,494	638	1.43	0.001	61.88	7.87	2.0
Zone*Cape*Year	35	13,968	399	1.07	0.299	10.66	3.27	0.3
Zone*Site[Cape]*Year	46	17,110	372	0.83	0.953	-53.33	-7.30	-1.7
**Res**	211	94,172	446			446.31	21.13	14.5
**Total**	462	790,780				3073.18		

b) SIMPER For Within-Zone Similarity					
**Taxon**	**Av.Abund**	**Av.Sim**	**Sim/SD**	**Contrib%**	**Cum.%**
**High Zone**					
Bare Space	3.58	16.77	3.23	26.98	26.98
*Balanus*	2.57	10.11	2.08	16.27	43.26
Herbivorous snails	3.21	8.62	0.98	13.87	57.13
Limpets	2.62	8.49	1.44	13.66	70.78
*Fucoid algae*	1.4	4.09	1.05	6.59	77.37
Chthamalus	1.16	3.88	1.87	6.25	83.62
Mussels	1	2.85	1.22	4.58	88.2
Mastocarpus	0.67	1.93	1.12	3.1	91.3
Endocladia	0.52	1.27	0.85	2.04	93.35
Algal crusts	0.56	1.26	0.78	2.03	95.37
**Mid Zone**					
Mussels	4.05	26.13	2.74	42.71	42.71
Limpets	3.3	15.62	1.79	25.53	68.23
Predatory snails	1.35	4.59	1.07	7.5	75.73
Bare Space	1.13	4.21	1.33	6.88	82.61
Large barnacles	0.92	3.05	1.16	4.98	87.59
Gooseneck barnacles	0.79	2.31	0.79	3.78	91.37
Balanus	0.5	1.28	0.81	2.09	93.46
Chthamalus	0.31	0.8	0.76	1.3	94.76
Anemones	0.21	0.53	0.69	0.86	95.62
Endocladia	0.3	0.49	0.3	0.8	96.42
**Low Zone**					
Bare Space	2.83	9.87	3.29	18.14	18.14
Articulated coralline algae	2.27	7.38	2.48	13.56	31.7
*Coralline algae crusts*	1.56	4.21	1.59	7.73	39.43
Limpets	1.66	3.72	1.12	6.84	46.27
Hedophyllum	1.36	2.98	0.97	5.47	51.74
Fine branching reds	1.09	2.78	1.37	5.1	56.84
Mazzaella blades	1.08	2.68	1.28	4.92	61.76
Surf grass	1.05	2.08	0.8	3.82	65.58
Anemones	0.87	1.9	0.94	3.5	69.08
Algal crusts	0.83	1.9	1.14	3.49	72.57

*Community associations with space and time*. To investigate the relative importance of temporal and spatial (among-site) variation on community structure, we tested the effects of cape, site (nested within cape), year, and their interactions (all as random effects) on community structure by zone using PERMANOVA. Here again the large size of the low zone dataset prevented quadrat-level model runs for PERMANOVAs and made visualizing trends in nMDS plots difficult. Thus, we averaged each functional group by transect for all low zone PRIMER analyses. Mid and high zone analyses were performed at the quadrat level since transect level averaging severely reduced model degrees of freedom. Relative importance (see above for initial percent contribution calculation for each term) of the spatial and temporal components of variation were estimated as: spatial % contribution = Σ(cape + site + 0.5*(year x cape) + 0.5*(year x site), and temporal % contribution = Σ(year + 0.5*(year x cape) + 0.5*(year x site] [89].

*Community associations with oceanic climate*. We used PERMANOVA to investigate if changes in community structure over time in each zone were correlated with the focal climate indices that the organisms would have been subjected to over the prior year (i.e. between surveys). Because environmental data were on different scales, we normalized them (ranging from -2 to +2) before analysis using Primer 7 [88] with the PERMANOVA+ add-on [89]. We inspected draftsman plots and found no substantial collinearity among the 8 environmental variables that might interfere with models (All R^2^ values < 0.4, except R^2^ = 0.523, 0.456, 0.410, 0.404 for PDO vs. SST, PDO vs. ENSO, NPGO vs. Year since SSWD and BEUTI vs. SD BEUTI, respectively). In each model, we included cape and site (nested within cape) as random factors and the eight climate indices as fixed factors (mean PDO, mean NPGO, mean ENSO, mean BEUTI, SD BEUTI, maximum air temperature, mean sea surface temperature, and years since SSWD). To identify candidate functional groups that may have been associated with environmental changes, we used SIMPER to identify the 10 most temporally variable functional groups by zone (highest average dissimilarity among years). We then analyzed their sensitivity to annual environmental fluctuations with univariate generalized linear mixed models (GLMMs) using the *glmer* function in the *lme4* package in R [94]. Site was random and climate indices were fixed. We used a Bonferroni correction threshold of p <0.00625 (8 terms). For significant climate index terms, we modeled and graphed the bivariate correlation between the cover or density of the taxon and the climate variable at the transect level (using the *lm()* function in the *stats* package and the *ggplot2* package [95]).

*Temporal trajectories of community structure*. Because strong effects of site and cape made temporal patterns difficult to distinguish in nMDS space, we calculated centroids of Bray-Curtis similarity for each site and year, then plotted year to year trajectories using nMDS and saved the coordinates. We used the vector lengths of these trajectories (quantified using as.ltraj function in the adehabitatLT package in R [96]) to calculate annual rates of community shifts [11,97]. For each zone, we then tested if the log-transformed rates of community shifts differed 1) among years, with cape and sites (nested within cape), as random variables, and 2) with the 8 climate variables, with site as a random variable (lmer function in lme4 package [94]).

*Taxon diversity*. Diversity analyses used the Shannon diversity index (H’ calculated using natural log of species or taxon cover to the highest possible resolution) for each transect. We tested whether taxon diversity varied among sites, years and their interaction using 3 linear models (one per zone, lm() function in stats package [90]). Next, we analyzed whether diversity in each zone varied with site (as a random factor) and the 8 climate indices using linear mixed models (lmer function in the lme4 package [94]). For significant climate variable terms (Bonferroni cutoff at P = 0.002), we regressed the focal climate indices on taxon diversity.

## Results

### Environmental trends

**Fig 2:**
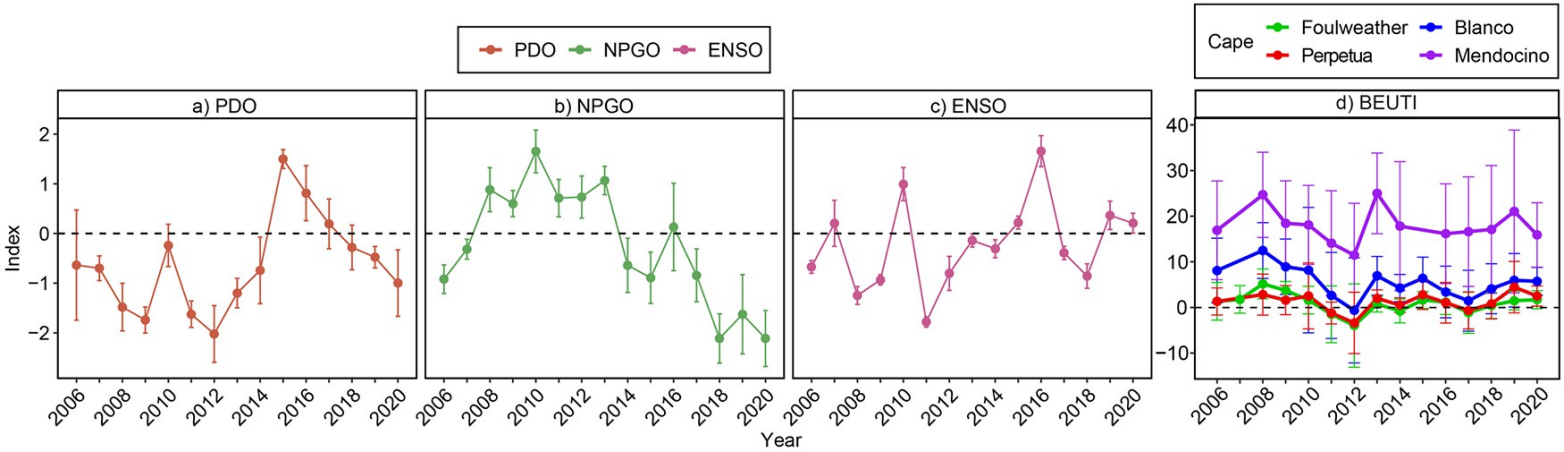
Average yearly environmental indices (Mean ± SD) for a) Pacific Decadal Oscillation (PDO), b) North Pacific Gyre Oscillation (NPGO), c) Multivariate El Niño Southern Oscillation Index (ENSO) and d) Biologically Effective Upwelling Transport Index, colored by Cape (BEUTI). All yearly means were calculated from daily or monthly indices during seasonal peak months for each variable, with PDO index from October—March, NPGO index from December—March, ENSO from December—April, and BEUTI from March through the survey month. These data were included in models analyzing community structure.

Temporal patterns of climate indices varied considerably during the study period (Fig 2). For example, the PDO mostly was negative (cooler conditions) from 2006 to 2013, then positive (warmer) in 2015 to 2017 while gradually declining then moving to negative in 2018 to 2020 (Fig 2a). The NPGO shifted from negative (calmer conditions) in 2006 to 2007 to positive (windier) from 2008 to 2013, then switched to negative and generally declined from 2014 to 2020 (Fig 2b). Finally, the ENSO index oscillated between cooler La Niña (2008, 2009, 2011) and warmer El Niño (2010, 2016) conditions (Fig 2c). Upwelling mostly varied latitudinally, as expected, with mean upwelling being similar at the two northern capes (Capes Foulweather and Perpetua), stronger in southern Oregon (Cape Blanco), and strongest in northern California (Cape Mendocino). It tended to decline from 2008 to 2012, spiked in 2013, then varied little annually after that (Fig 2d).

**Fig 3:**
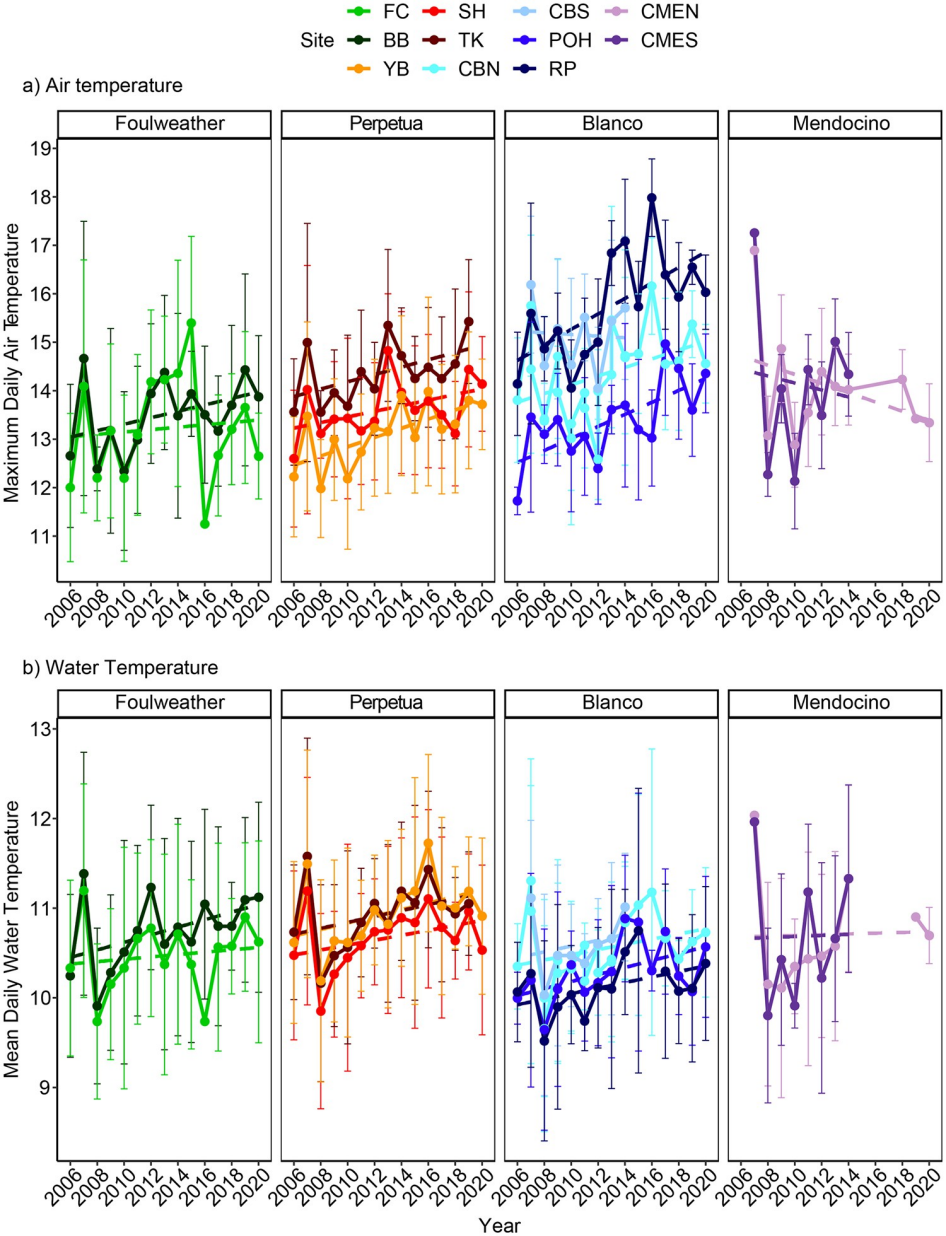
Average yearly measures (Mean ± SD) among capes (panels) and sites (colors) for a) the maximum daily air temperature (Max Air Temp), and b) the mean daily water temperature (Mean SST). Cape Foulweather (greens) includes Boiler Bay (BB) and Fogarty Creek (FC), Cape Perpetua (reds) includes Yachats Beach (YB), Strawberry Hill (SH) and Tokatee Klootchman (TK), Cape Blanco (blues) includes Cape Blanco North(CBN), Cape Blanco South (CBS), Port Orford Heads (POH) and Rocky Point (RP), and Cape Mendocino (purples) includes Cape Mendocino North (CMEN) and South (CMES). Dashed lines are linear fits of the data. All daily values were calculated from loggers recording every 15 minutes, then the yearly means and standard deviations were calculated from daily values. Air temperature values were taken from warm seasons (from April to the survey month) and water temperature was taken for the year. Missing data are from years where we were unable to maintain or discontinued the instrument deployments. These data were included as covariates in models analyzing community structure.

For temperature trends, both mean maximum air temperature (Fig 3a) and mean water temperature (Fig 3b) generally increased during the study period. The strongest increases occurred for maximum daily air temperature at Rocky Point, Port Orford Heads, Cape Blanco North, and Yachats Beach (P < 0.01, with R^2^ = 0.22. 0.12, 0.07, and 0.09, respectively). As reported earlier [12], a period of elevated water temperature occurred during the confluence of the 2014–16 marine heat wave and the 2015–16 El Niño (Fig 3b). As expected, warmer water temperature was correlated to stronger PDO (R^2^ = 0.523, P = < 0.001), but no other correlations of temperatures with climate oscillations were significant. Mean daily water temperatures were similar between capes and between sites (Fig 3b). Maximum daily air temperatures were generally warmer at Cape Blanco that the other capes, and air temperatures were fairly similar among sites within capes (Fig 3a). The exception was Cape Blanco, where maximum daily air temperatures were typically lower at Port Orford Heads and higher at Rocky Point and Cape Blanco South compared to the Cape Blanco North site.

### Spatial versus temporal community variation

**Fig 4:**
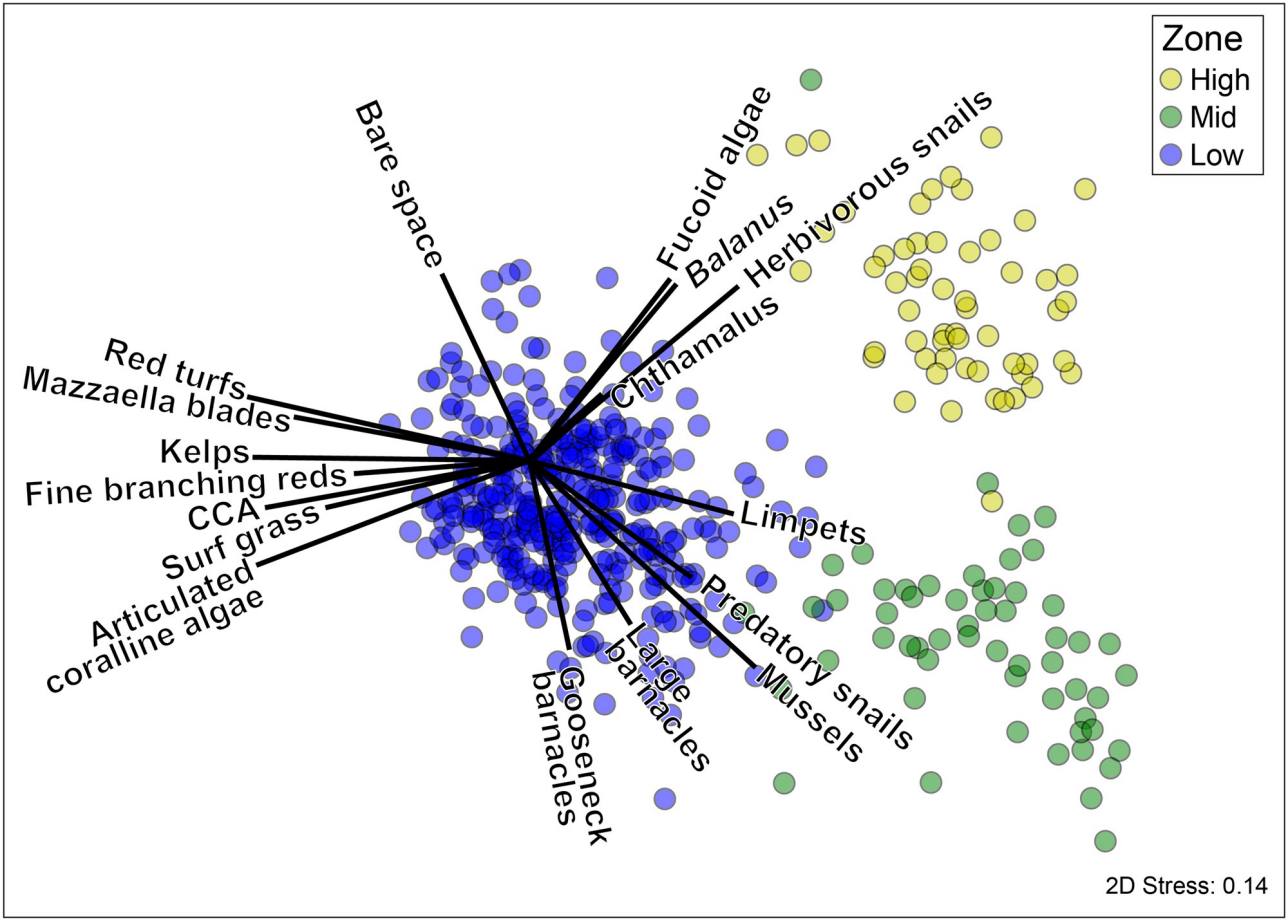
Non-metric multidimensional scaling (nMDS) plots depicting Bray-Curtis dissimilarity in community structure among high (yellow), mid (green), and low (blue), and intertidal zones. Vector overlays depict species contributing >3% to within-zone similarity in SIMPER analyses (S2b Table in S1 Appendix). Each data point represents a community at the transect level (the average of ~ten 0.5 x 0.5m quadrats).

**Fig 5:**
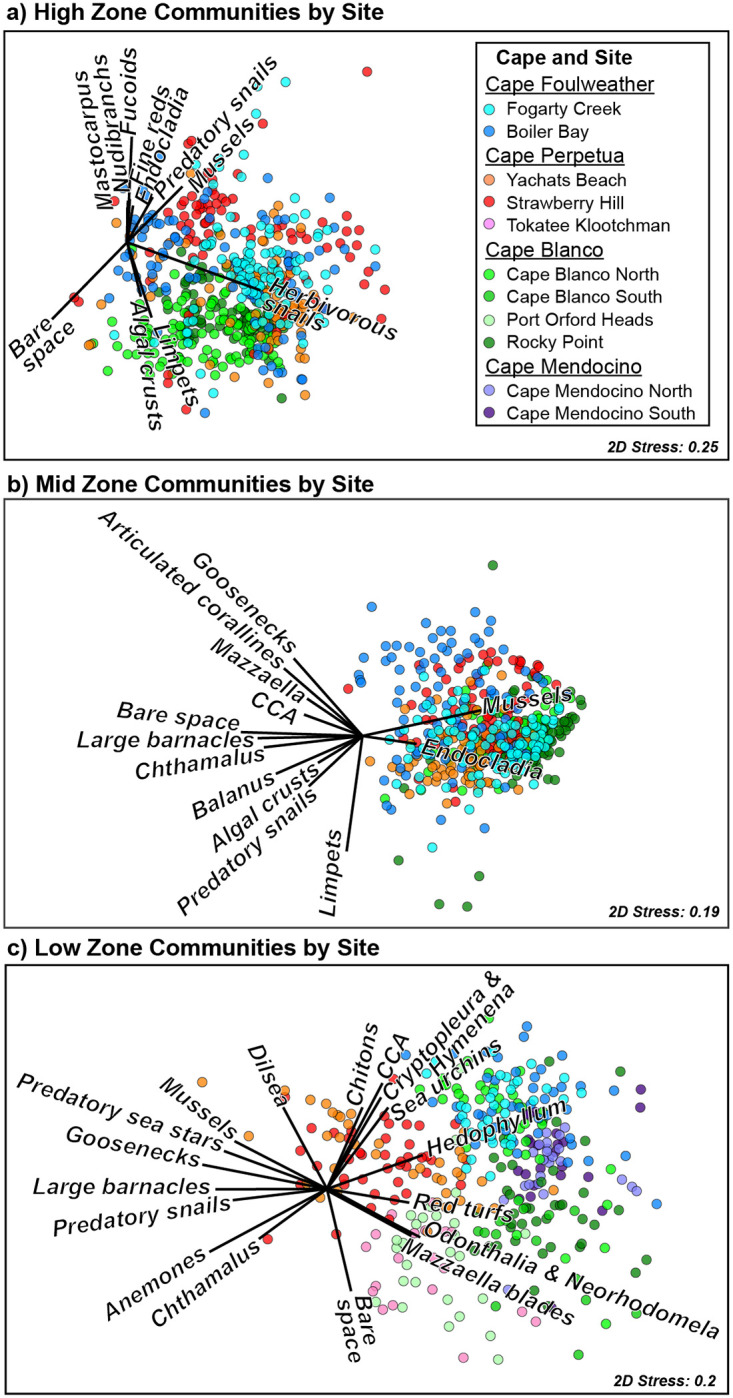
Non-metric multidimensional scaling (nMDS) plots depicting Bray-Curtis dissimilarity among intertidal communities in a) high, b) mid, and c) low intertidal community structure in Oregon and northern California from 2006–2021. Colors depict Cape Foulweather sites in blues, Cape Perpetua sites in reds, Cape Blanco sites in greens, and Cape Mendocino sites in purples. Each data point represents a community at either the quadrat level (a & b, 0.5 x 0.5m) or transect level (c, the average of ~10 0.5 x 0.5m quadrats). Vector overlays (in black) depict species contributing most strongly to within-cape similarity in SIMPER analyses (S4d-S4f Table in S1 Appendix).

**Fig 6:**
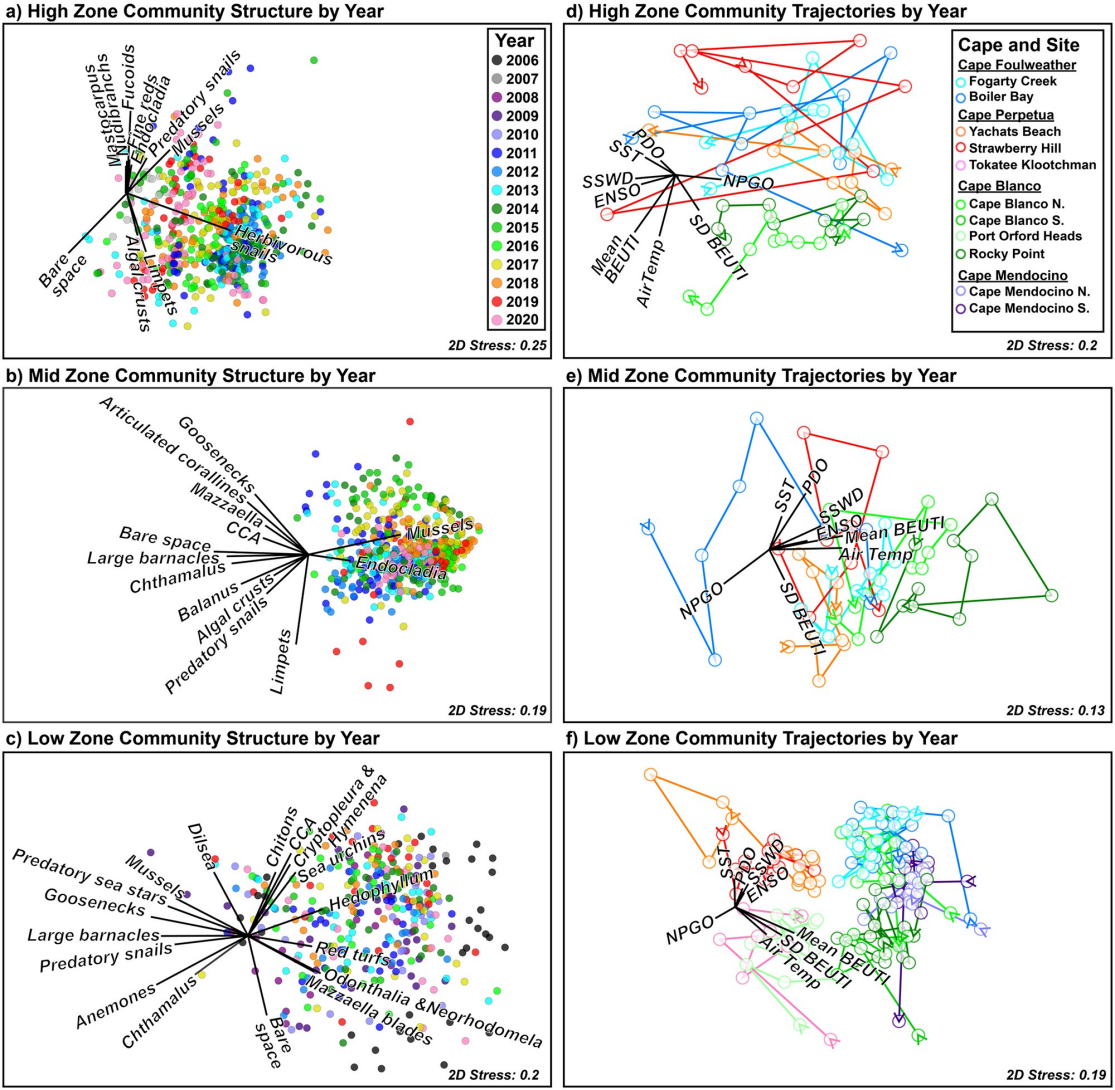
Non-metric multidimensional scaling (nMDS) plots depicting Bray-Curtis dissimilarity among intertidal communities in high (a & d), mid (b & e), and low (c & f) intertidal community structure in Oregon and northern California from 2006–2021. Left panels (a-c) depict dissimilarities among years, with each data point representing a community at either the quadrat level (a & b, 0.5 x 0.5m) or transect level (c, the average of ~10 0.5 x 0.5m quadrats). Left panel (a-c) vector overlays (in black) depict species contributing most strongly to within-cape similarity in SIMPER analyses (S4d-S4f Table in S1 Appendix). Right panels (d-e) depict community trajectories over time with each data point representing the centroid of community structure for each site and year (i.e. centroids from left panels). We calculated the length and absolute angle of these trajectories to estimate rate and direction of community shifts (respectively) for subsequent analyses. Right panel (d-e) vector overlays depict the association between the environmental variables and community dissimilarity. Right panel (d-e) colors depict Cape Foulweather sites in blues, Cape Perpetua sites in reds, Cape Blanco sites in greens, and Cape Mendocino sites in purples.

Community structure was more strongly associated with spatial (zone, cape and site) than temporal variables (year) (Table 1). As expected, zones were clearly separated visually (Fig 4), as were capes and often sites (Fig 5). However, visual separation among years was much less clear (Fig 6a–6c). Within each zone, cape and site were associated with more community variation than was year (S3a-S3c Table in S1 Appendix; High: 35.6% vs. 19.9%, Mid: 33.2% vs. 19.4%, and Low: 48.4% vs. 14.7%, for spatial vs. temporal variation, see calculation in methods).

### Community structure among intertidal zones

As indicated above, community structure was strongly associated with intertidal zone, which contributed 55.3% of model fit when analyzing the contributions of zone, cape, site and year (Fig 4, Table 1; PERMANOVA: Zone: p = 0.001). In SIMPER analyses, the strongest drivers of within-zone community similarity (i.e., contributing >5%) for the high zone were bare space, barnacles, herbivorous gastropods, and fucoid algae (Fig 4; S3d Table in S1 Appendix; S1 Fig in S3 Appendix). In the mid zone, mussels, limpets, whelks, bare space, and large barnacles drove community similarity (Fig 4; S3e Table in S1 Appendix; S2 Fig in S3 Appendix). The low zone was typified by bare space, articulated coralline algae, crustose coralline algae, limpets, the kelp *Hedophyllum sessile*, and fine branching red algae (Fig 4; S3f Table in S1 Appendix; S3 Fig in S3 Appendix).

### Geographic variation in community structure

Taxon abundance often differed by cape and site (Fig 5). Although community structure did not differ by cape in the high zone (Fig 5a; S3a Table in S1 Appendix) and functional group relative abundance was similar among capes (S3d Table in S1 Appendix), community structure differed among sites within capes (Fig 5a; p < 0.007 for all site comparisons). These trends suggest that high zone variation was controlled at a localized scale.

In the mid zone, among-cape differences (Fig 5b; S3b Table in S1 Appendix) were driven by separation of Cape Blanco from Capes Foulweather and Perpetua (p = 0.023, p = 0.014, respectively), which did not differ from each other (p = 0.311). As in the high zone, sites differed within capes (Fig 5b; S3b Table in S1 Appendix; p < 0.018 for site comparisons). While dominant mid-zone species were often similar among capes, the strongest contributors to community separation among capes were mussels and limpets at Cape Blanco (Fig 5b; S3e Table in S1 Appendix; 58.9% and 31.2% contribution, respectively), predatory snails and bare space at Cape Perpetua (17.0% and 6.3% contribution, respectively) and gooseneck barnacles at Cape Foulweather (5.9% contribution). In the low zone, most capes differed from one another (Fig 5c; S3c Table in S1 Appendix) except Cape Blanco did not differ from Capes Foulweather or Mendocino (p = 0.091 and 0.089 respectively). Sites again differed within capes (p < 0.032 for site comparisons). Like the other zones, in the low zone many of the same taxa dominated every cape, but their relative contributions to cape separation differed (Fig 5c; S3f Table in S1 Appendix). For example, Cape Foulweather was associated with relatively low bare space but with high coralline algae crusts, *Hedophyllum sessile*, and *Cryptopleura ruprechtiana/ Hymenena flabelligera* (Fig 5c; S3f Table in S1 Appendix, 11.4%, 10.9%, 7.7%, and 6.1% contribution, respectively) while Cape Perpetua had relatively more sea anemones, predatory sea stars, and gooseneck barnacles (10.0%, 5.1% and 5.0% contribution, respectively).

### Community structure over time

Interannual differences occurred in all zones, with year accounting for 19.9%, 19.4%, and 14.7% of the community variation in high, mid and low zones, respectively (S3a-S3c Table in S1 Appendix; Year: p = 0.001 for each zone, see % calculation in methods). However, these differences were not readily apparent in nMDS visualizations (Fig 6a–6c) and the temporal patterns varied with site in all zones and with cape in the low zone (S3a-S3c Table in S1 Appendix). In the high zone, only 2007 differed from each of the other years (p < 0.005), but this was because only Boiler Bay was sampled that year. In the mid zone, only 2012 differed from 2017 and 2018 (p = 0.038 and 0.044, respectively). On the other hand, in the low zone several years differed from one another, particularly 2006, 2008, and 2019 compared to other years.

### Community associations with oceanic climate

#### Community and functional group patterns

In all zones, all oceanic climate fluctuations were related to community structure (p < 0.03 in most cases), but with the exception of years since sea star wasting disease in the mid zone (15.9% of variance explained), the amount of variance explained by each was low (ranging from 0.6 to 8.6%; Table 2). In the high zone, no single factor accounted for more than 3.8% of the variation in community structure, and together the 8 factors drove only 16.1% of the variation in community structure (Table 2a). In the mid zone, environment-community associations were stronger (Table 2b, p < 0.012). Collectively, the climate indices, upwelling and temperature accounted for 25.5% of the variance, with NPGO and PDO contributing the most strongly (8.6% and 6.1% of variation, respectively). As noted above, the years since sea star wasting accounted for a further 15.9% of the variance explained, bringing the total variance explained by environment to 41.3% in the mid zone. In the low zone, all factors except upwelling variation and maximum daily air temperature varied with community structure, but individual effects were weak with sea star wasting disease, NPGO and mean upwelling being the strongest drivers (Table 2c, 6.2%, 4.5%, and 4.2%, respectively). Collectively, environment explained only 20.1% of the low zone variance.

**Table 2:** Results of 3 PERMANOVA analyses testing drivers of community dissimilarity in a) high, b) mid, and c) low intertidal zones in Oregon and northern California from 2006–2021. We tested the effects of site (nested within cape) as a random factor and the effects of climate variables for mean Pacific Decadal Oscillation index (mean PDO), mean North Pacific Gyre Oscillation index (mean NPGO), mean El Niño Southern Oscillation index (mean ENSO), mean and standard deviation of the Biologically Effective Upwelling Transport Index (mean and SD BEUTI, respectively), mean daily sea surface temperature (Mean SST), mean daily maximum air temperature (Max Air Temp), and years since the sea star wasting disease epidemic (Yr Since SSWD). Communities were analyzed at the quadrat level in the mid and high zones (0.5 x 0.5m) and the transect level in the low zone (the average of ~10 0.5 x 0.5m quadrats). Bold statistics indicate that >10% of variation was explained by a given factor.

**a) High Zone**								
**Source**	**df**	**SS**	**MS**	**Pseudo-F**	**P**	**Estimate**	**Sq.root**	**% Explained**
**Mean PDO**	1	7,842	7,842	8.92	0.001	55.0	7.41	3.8
**Mean NPGO**	1	7,583	7,583	8.62	0.001	55.1	7.42	3.8
**Mean ENSO**	1	6,526	6,526	7.42	0.001	32.5	5.70	2.2
**Mean BEUTI**	1	5,020	5,020	5.71	0.001	31.2	5.58	2.2
**SD BEUTI**	1	1,900	1,900	2.16	0.056	4.6	2.14	0.3
**Mean SST**	1	3,570	3,570	4.06	0.001	23.2	4.81	1.6
**Max Air Temp**	1	2,312	2,312	2.63	0.018	8.9	2.98	0.6
**Yr Since SSWD**	1	3,124	3,124	3.55	0.004	22.1	4.70	1.5
**Site**	5	121,360	24,272	27.60	0.001	333.6	18.27	**23.1**
**Residual**	560	492,460	879			879.4	29.65	60.8
**Total**	573	742,780				1445.4		
**b) Mid Zone**								
**Source**	**df**	**SS**	**MS**	**Pseudo-F**	**P**	**Estimate**	**Sq.root**	**% Explained**
**Mean PDO**	1	12,885	12,885	15.40	0.001	112.0	10.59	6.1
**Mean NPGO**	1	6,593	6,593	7.88	0.001	157.4	12.54	8.6
**Mean ENSO**	1	5,797	5,797	6.93	0.001	71.7	8.47	3.9
**Mean BEUTI**	1	5,090	5,090	6.08	0.001	43.9	6.63	2.4
**SD BEUTI**	1	6,355	6,355	7.60	0.001	35.1	5.93	1.9
**Mean SST**	1	2,742	2,742	3.28	0.012	28.6	5.35	1.6
**Max Air Temp**	1	3,296	3,296	3.94	0.002	16.3	4.04	0.9
**Yr Since SSWD**	1	8,274	8,274	9.89	0.001	292.2	17.09	**15.9**
**Site**	5	84,260	16,852	20.14	0.001	239.4	15.47	**13.1**
**Residual**	541	452,660	837			836.7	28.93	45.6
**Total**	554	663,550				1833.4		
**c) Low Zone**								
**Source**	**df**	**SS**	**MS**	**Pseudo-F**	**P**	**Estimate**	**Sq.root**	**% Explained**
**Mean PDO**	1	1,187	1,187	2.03	0.032	7.1	2.66	0.6
**Mean NPGO**	1	8,489	8,489	14.52	0.001	56.4	7.51	4.5
**Mean ENSO**	1	4,851	4,851	8.3	0.001	32.9	5.74	2.6
**Mean BEUTI**	1	3,065	3,065	5.24	0.001	53.2	7.3	4.2
**SD BEUTI**	1	587	587	1	0.43	0	0.13	0
**Mean SST**	1	2,579	2,579	4.41	0.001	23	4.79	1.8
**Max Air Temp**	1	730	730	1.25	0.252	1.5	1.21	0.1
**Yr Since SSWD**	1	8,977	8,977	15.35	0.001	78.6	8.87	6.2
**Site**	10	112,240	11,224	19.19	0.001	422.1	20.55	**33.5**
**Residual**	317	185,370	585			584.8	24.18	46.4
**Total**	335	365,900				1259.6		

**Fig 7:**
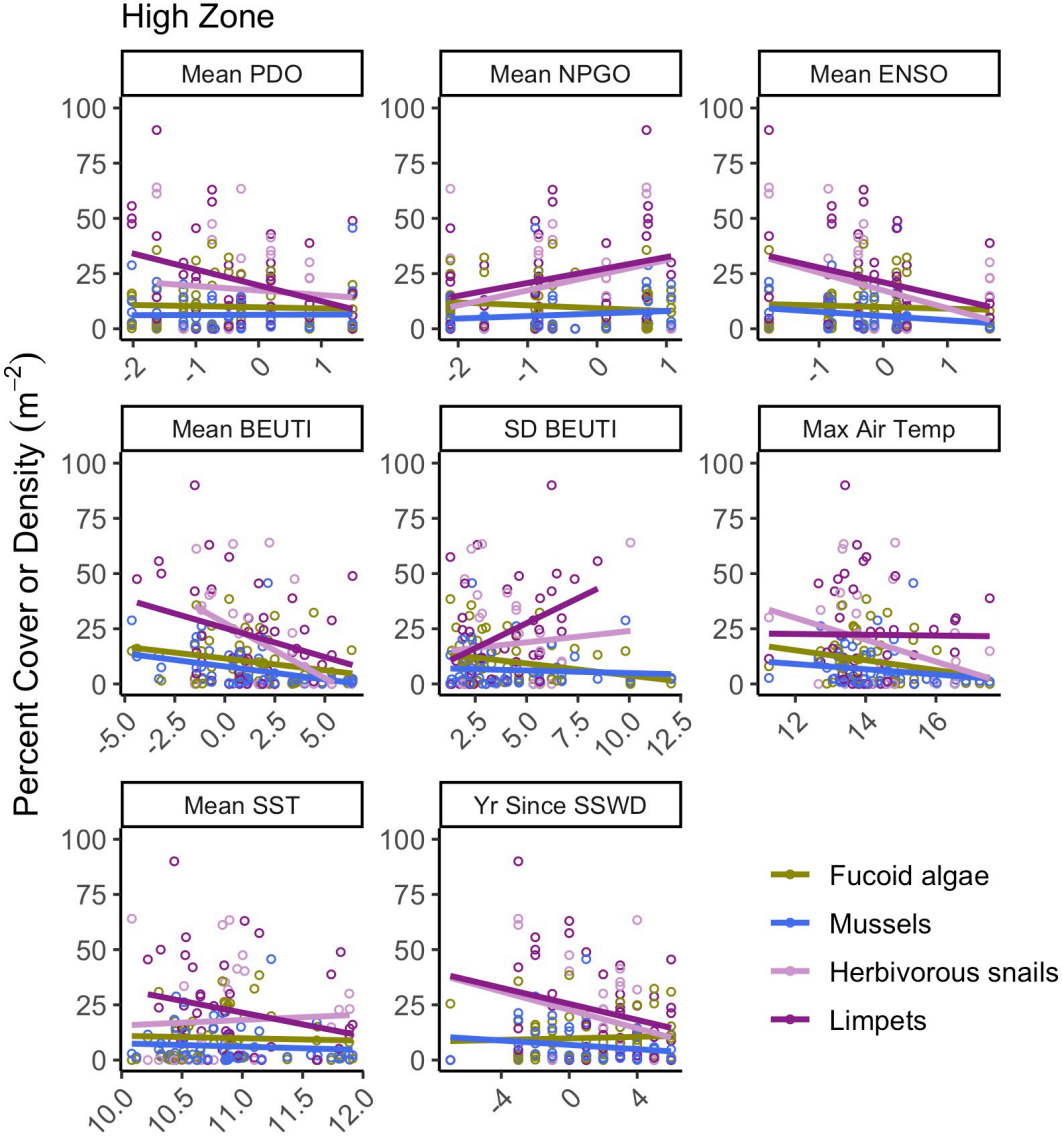
Bivariate regressions between the percent cover of focal functional groups (significant functional groups in Table 2a with the 8 focal climate variables in the high zone. Each data point is the average abundance of a functional group for a site and year. Environmental drivers included Pacific Decadal Oscillation index (mean PDO), North Pacific Gyre Oscillation index (mean NPGO), Multivariate El Niño Southern Oscillation Index (mean ENSO), Biologically Effective Upwelling Transport Index (mean BEUTI), the standard deviation in BEUTI (SD BEUTI), the maximum daily air temperature in celcius by site (Max Air Temp), the mean daily water temperature in celcius by site (Mean SST) and the years since the initial sea star wasting disease outbreak (Yrs since SSWD). Colors correspond to functional groups, with fucoid algae in green, mussels (Mytilus spp.) in in blue, herbivorous snails in pink, and limpets in purple. Units for fucoid algae and mussels are in percent cover while limpets and herbivorous snails are in density m-2. Some high densities (100-400m-2) of limpets are not shown in plots to enable visualizations of fits.

Analysis of correlations between the most temporally variable functional groups (S4, S5 and S6 Figs in S3 Appendix) and the 8 oceanic environmental variables showed that several groups appeared sensitive to the environment in the high and mid zones, but fewer were sensitive in the low zone. In the high zone, both herbivorous snails and limpets were sensitive to several environmental variables (Fig 7; Table 3a), and the strongest trends were increases with windier phases of the NPGO (R^2^ = 0.157 and 0.122, respectively), decreases with years since SSWD (R^2^ = 0.239 and 0.151, respectively), and limpet abundance increased with higher upwelling variability (R^2^ = 0.164). Finally, fucoid algal abundance in the high zone was weakly negatively correlated with upwelling variability and mussel abundance was weakly positively correlated with PDO (Fig 7; Table 3a; R^2^ = 0.077 and < 0.001, respectively).

**Table 3:** Analysis results for the focal functional groups with the highest average annual dissimilarity in the a) high, b) mid and c) low zone. Multiple regression models tested the effects of mean Pacific Decadal Oscillation index (mean PDO), mean North Pacific Gyre Oscillation index (mean NPGO), mean El Niño Southern Oscillation index (mean ENSO), mean and standard deviation of the Biologically Effective Upwelling Transport Index (mean and SD BEUTI, respectively), mean daily sea surface temperature (Mean SST), mean daily maximum air temperature (Max Air Temp), and years since the sea star wasting disease epidemic (Yr Since SSWD) Only those model terms that met the significance threshold of P < 0.00625 (Bonferroni correction) are listed, and these were subsequently tested for bivariate correlations between the climate term and the taxon abundance. Bold statistics indicate bivariate adjusted R^2^ of > 0.1.

**a) High zone**				
**Model Response**	**Average Annual Dissimilarity**	**Model Marginal R** ^**2**^	**Sig. Terms (p < 0.0065)**	**Bivariate Correlation**
**Herbivorous snails**	8.40	0.34	Mean PDO	-0.057
			Mean NPGO	**+0.157**
			ENSO	-0.054
			SD BEUTI	-0.001
			Max Air Temp	-0.048
			Mean SST	< -0.001
			Yr Since SSWD	**-0.239**
**Limpets**	6.45	0.42	Mean PDO	-0.038
			Mean NPGO	**+0.122**
			ENSO	-0.037
			BEUTI	+0.044
			SD BEUTI	**+0.164**
			Max Air Temp	+0.059
			Mean SST	-0.029
			Yr Since SSWD	**-0.151**
***Balanus glandula***	4.55	0.05	None sig.	
**Fucoid algae**	4.46	0.51	SD BEUTI	-0.077
***Chthamalus dalli***	3.12	0.12	None sig.	
**Mussels**	3.10	0.32	PDO	<+0.001
**Bare Space**	2.74	0.13	None sig.	
***Mastocarpus papillatus***	2.35	0.18	None sig.	
**b) Mid zone**				
**Model Response**	**Average Annual Dissimilarity**	**Model Marginal R2**	**Sig. Terms (p < 0.0065)**	**Bivariate Correlation**
**Limpets**	7.98	0.40	Mean PDO	-0.222
			Mean NPGO	**+0.107**
			Mean ENSO	-0.025
			SD BEUTI	+0.109
			Max Air Temp	-0.005
			Mean SST	-0.065
			Yr Since SSWD	**-0.131**
**Predatory Snails**	5.13	0.39	Mean NPGO	+0.030
			Mean ENSO	<+0.001
			SD BEUTI	-0.034
			Max Air Temp	**-0.117**
			Yr Since SSWD	-0.012
**Bare Space**	4.74	0.32	None sig.	
**Large barnacles**	4.37	0.33	None sig.	
**Gooseneck barnacles**	3.63	0.27	Mean NPGO	-0.010
			Yr Since SSWD	-0.001
**Mussels**	2.71	0.22	Yr Since SSWD	**+0.136**
***Endocladia muricata***	2.30	0.63	Max Air Temp	**+0.255**
***Balanus glandula***	2.18	0.60	None sig.	
**c) Low zone**				
**Model Response**	**Average Annual Dissimilarity**	**Model Marginal R2**	**Sig. Terms (p < 0.0065)**	**Bivariate Correlation**
**Limpets**	2.93	0.52	Mean PDO	-0.088
			Mean NPGO	0.059
			Mean ENSO	-0.097
			BEUTI	0.001
			SD BEUTI	-0.004
			Max Air Temp	-0.003
			Mean SST	**-0.123**
			Yr Since SSWD	-0.050
**Surf grass**	2.61	0.09	Mean PDO	0.009
			Mean NPGO	0.007
			SD BEUTI	**-0.111**
***Hedophyllum sessilis***	2.51	0.06	Mean SST	-0.030
**Crustose Coralline Algae**	2.12	0.20	None sig.	
***Cryptopleura* and *Hymenena***	2.08	0.11	None sig.	
***Odonthalia* and *Neorhodomela***	1.94	0.05	None sig.	
**Bare Space**	1.77	0.08	Mean NPGO	0.009
			Mean ENSO	-0.018
			Max Air Temp	0.029
			Mean SST	-0.085
			Yr Since SSWD	-0.047
**Articulated Coralline Algae**	1.73	0.07	BEUTI	-0.027
			Max Air Temp	<0.001

**Fig 8:**
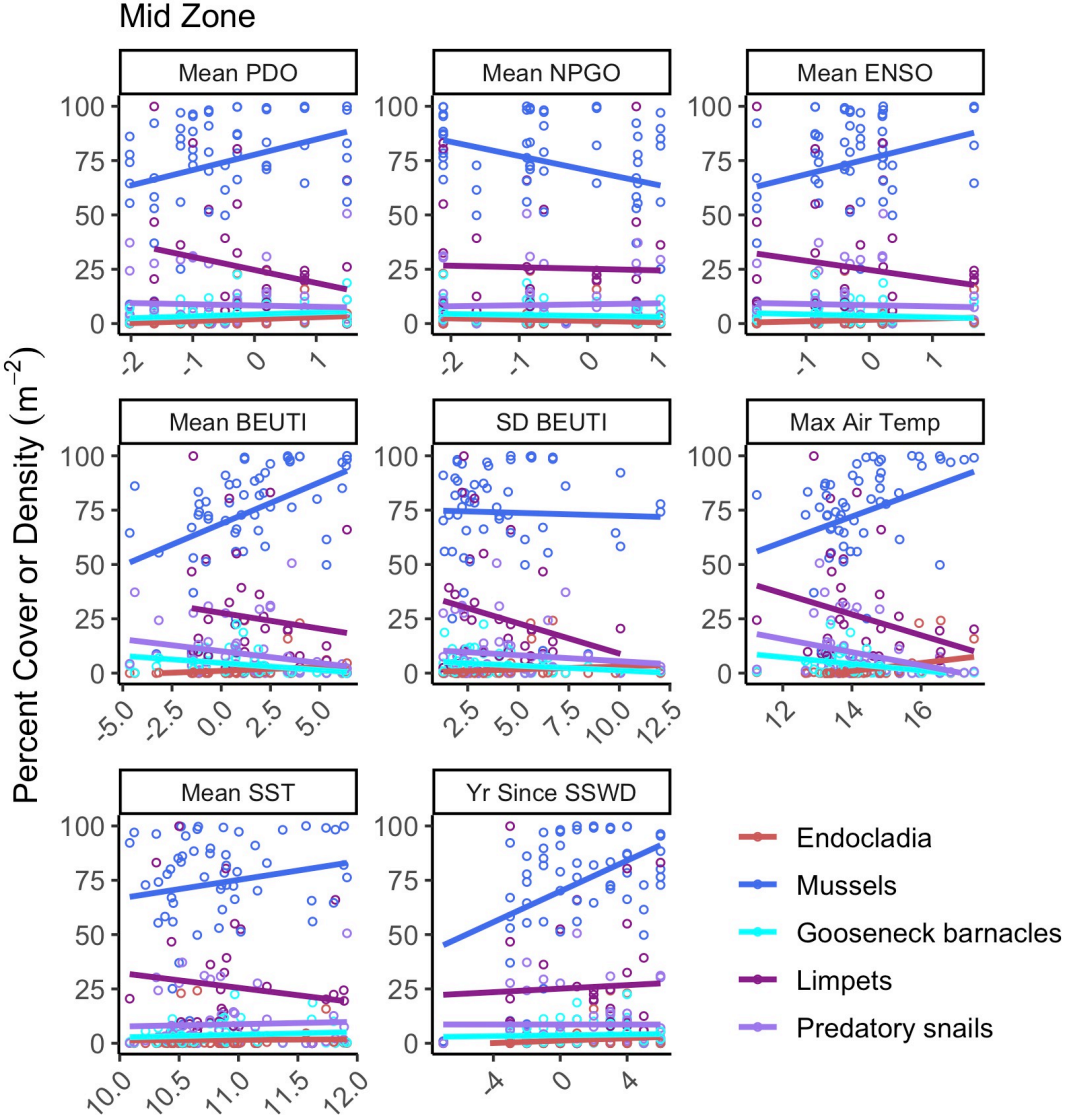
Bivariate regressions between the percent cover of focal functional groups (significant functional groups in Table 2b with the 8 focal climate variables in the mid zone. Each data point is the average abundance of a functional group for a site and year. Environmental drivers included Pacific Decadal Oscillation (mean PDO), North Pacific Gyre Oscillation (mean NPGO), Multivariate El Niño Southern Oscillation Index (mean ENSO), Biologically Effective Upwelling Transport Index (mean BEUTI), the standard deviation in BEUTI (SD BEUTI), the maximum daily air temperature by site (Max Air Temp), the mean daily water temperature by site (Mean SST) and the years since the initial sea star wasting disease outbreak (Yrs since SSWD). Colors correspond to functional groups, with *Endocladia muricata* in red, mussels (*Mytilus* spp.) in blue, gooseneck barnacles (*Pollicipes polymerus*) in cyan, limpets in dark purple, and predatory snails in light purple. Units are in percent cover except limpets and predatory snails are in density m-2.

**Fig 9:**
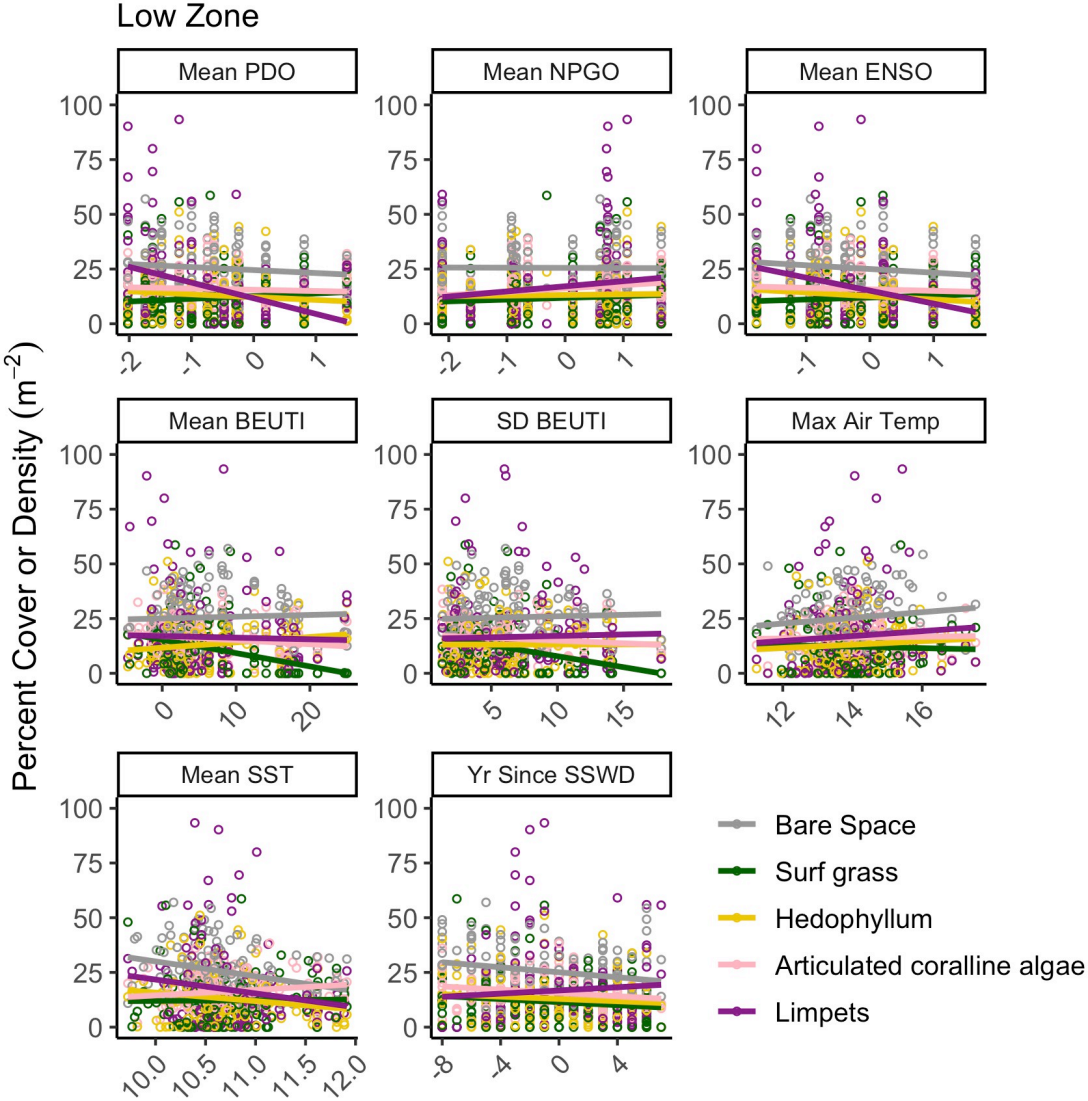
Bivariate regressions between the percent cover of focal functional groups (significant functional groups in Table 2c with the 8 focal climate variables in the low zone. Each data point is the average abundance of a functional group for a site and year. Environmental drivers included Pacific Decadal Oscillation (mean PDO), North Pacific Gyre Oscillation (mean NPGO), Multivariate El Niño Southern Oscillation Index (mean ENSO), Biologically Effective Upwelling Transport Index (mean BEUTI), the standard deviation in BEUTI (SD BEUTI), the maximum daily air temperature by site (Max Air Temp), the mean daily water temperature by site (Mean SST) and the years since the initial sea star wasting disease outbreak (Yrs since SSWD). Colors correspond to functional groups, with bare space in gray, surf grass (*Phyllospadix* spp.) in green, *Hedophyllum sessile* in yellow, articulated coralline algae in pink, and limpets in dark purple. Units are in percent cover except limpets are in density m^-2^.

Mid zone limpet abundance varied similarly to that in the high zone, increasing with windier NPGO and increased upwelling variation (R^2^ = 0.107 and 0.109, respectively), decreasing with years since SSWD (R^2^ = 0.131), and additionally decreasing in warmer phases of PDO (R^2^ = 0.222, Fig 8, Table 3b). Mid-zone predatory snail abundance was lower when maximum air temperature was warmer (R^2^ = 0.117), mussel abundance increased with years since SSWD (R^2^ = 0.136), and the alga *Endocladia muricata* was more abundant with increased maximum air temperature (R^2^ = 0.255, Fig 8, Table 3b). In the low zone, individual functional groups were generally less sensitive to varying environments (Fig 9, Table 3c), with the only notable trends being decreased limpets with warmer sea surface temperatures (R^2^ = 0.123) and decreased surfgrass (*Phyllospadix* spp.) with increased upwelling variability (R^2^ = 0.111).

#### Temporal trajectories of community structure

**Fig 10:**
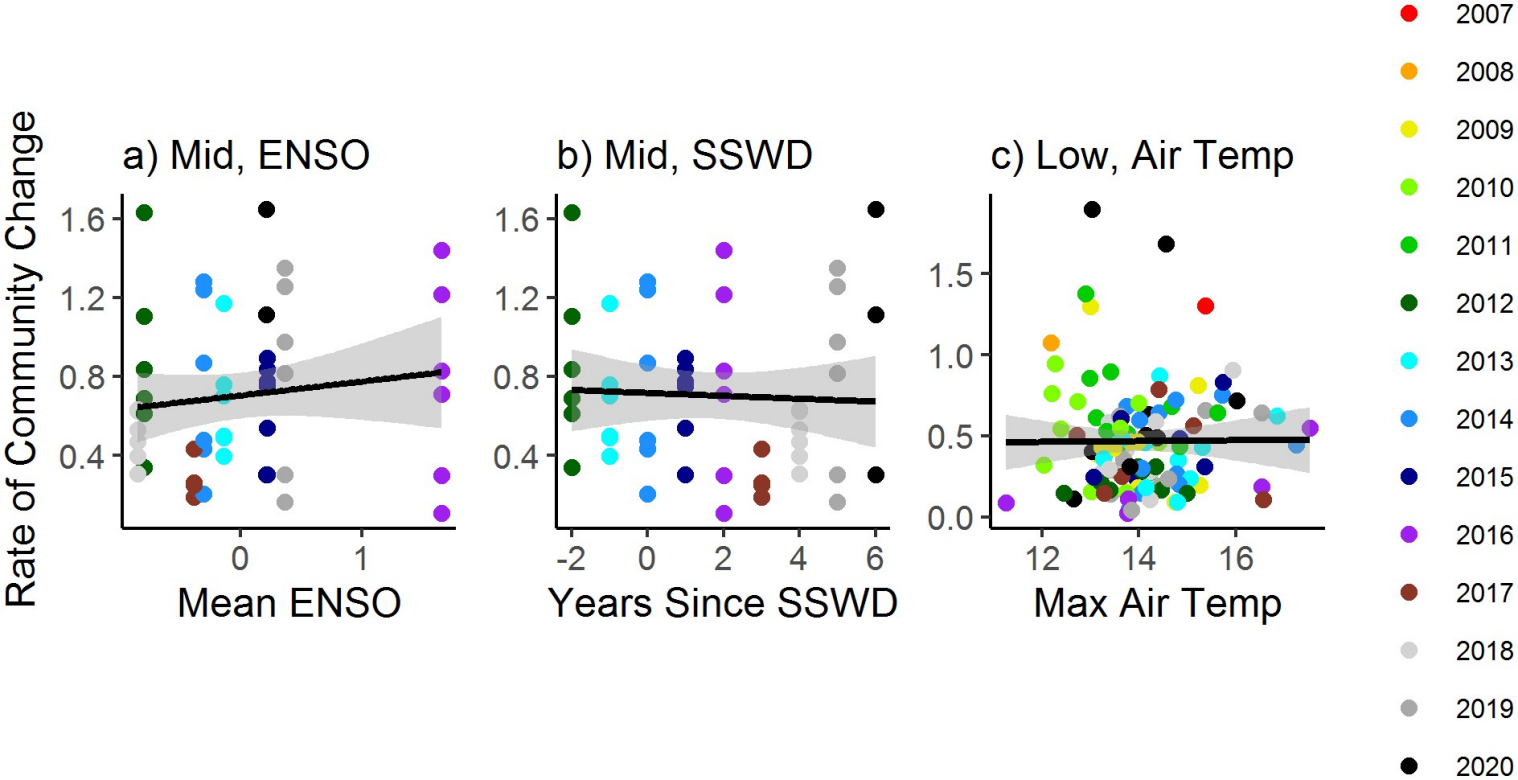
Correlations of the rate (vector lengths in Fig 4d–4f) of community change with significant environmental drivers (identified by models in Table 3) in Oregon and northern California from 2007–2020. Focal comparisons included the a) rate of change in the mid zone with Multivariate El Niño Southern Oscillation Index (mean ENSO) zone, b) rate of change in the mid zone with the years since the initial sea star wasting disease outbreak (Yrs since SSWD), and c) rate of change in the low zone with the maximum daily air temperature (Max Air Temp).

Trajectories of annual community shifts showed that communities did vary over time, but no consistent trend emerged within any zone or among years (Fig 6d–6f). In the high zone, the rate of community change was not correlated with any of the 8 environmental variables (Table 4a). In the mid zone, community change rates were weakly associated with mean ENSO index (R^2^ = -0.003; Fig 10a; Table 4b) and the years since sea star wasting disease (R^2^ = -0.019; Fig 10b; Table 4b). In the low zone, community change rate increased slightly with increasing maximum daily air temperature (R^2^ = 0.069; Fig 10c; Table 4c).

**Table 4:** Results of multiple regression analyses and bivariate regressions testing drivers of rate of community change in a) high, b) mid, and c) low intertidal zones in Oregon and northern California from 2006–2021. We tested the effects of peak seasonal climate indices for mean Pacific Decadal Oscillation index (mean PDO), mean North Pacific Gyre Oscillation index (mean NPGO), mean El Niño Southern Oscillation index (mean ENSO), mean and standard deviation of the Biologically Effective Upwelling Transport Index index (mean and sd BEUTI, respectively), mean daily sea surface temperature (Mean SST), mean daily maximum air temperature (Max Air Temp), and years since the sea star wasting disease epidemic (Yr Since SSWD). Communities were analyzed at the quadrat level in the mid and high zones (0.5 x 0.5m) and the transect level in the low zone (the average of ~10 0.5 x 0.5m quadrats). Bold statistics indicate significant terms (Bonferroni cutoff P < 0.002).

Factor	Chisq	Df	Pr(>Chisq)	Bivariate Adj R2
**a) High Zone**				
**Intercept**	0.76	1	0.3820	
**Mean PDO**	0.12	1	0.7339	
**Mean NPGO**	2.30	1	0.1298	
**Mean ENSO**	1.92	1	0.1653	
**Mean BEUTI**	2.25	1	0.1334	
**SD BEUTI**	3.33	1	0.0681	
**Mean SST**	0.14	1	0.7069	
**Max Air Temp**	0.91	1	0.3398	
**Yr Since SSWD**	3.33	1	0.0679	
**b) Mid Zone**				
**Intercept**	5.55	1	0.0185	
**Mean PDO**	0.03	1	0.8556	
**Mean NPGO**	8.34	1	0.0039	
**Mean ENSO**	9.71	1	**0.0018**	-0.003
**Mean BEUTI**	0.85	1	0.3557	
**SD BEUTI**	0.53	1	0.4681	
**Mean SST**	0.19	1	0.6612	
**Max Air Temp**	6.05	1	0.0139	
**Yr Since SSWD**	9.51	1	**0.0020**	-0.019
**c) Low Zone**				
**Intercept**	9.21	1	0.0024	
**Mean PDO**	4.46	1	0.0347	
**Mean NPGO**	1.77	1	0.1836	
**Mean ENSO**	0.55	1	0.4587	
**Mean BEUTI**	0.10	1	0.7575	
**SD BEUTI**	1.62	1	0.2037	
**Mean SST**	0.28	1	0.5953	
**Max Air Temp**	13.39	1	**0.0003**	0.069
**Yr Since SSWD**	2.74	1	0.0980	

### Taxon diversity

**Fig 11:**
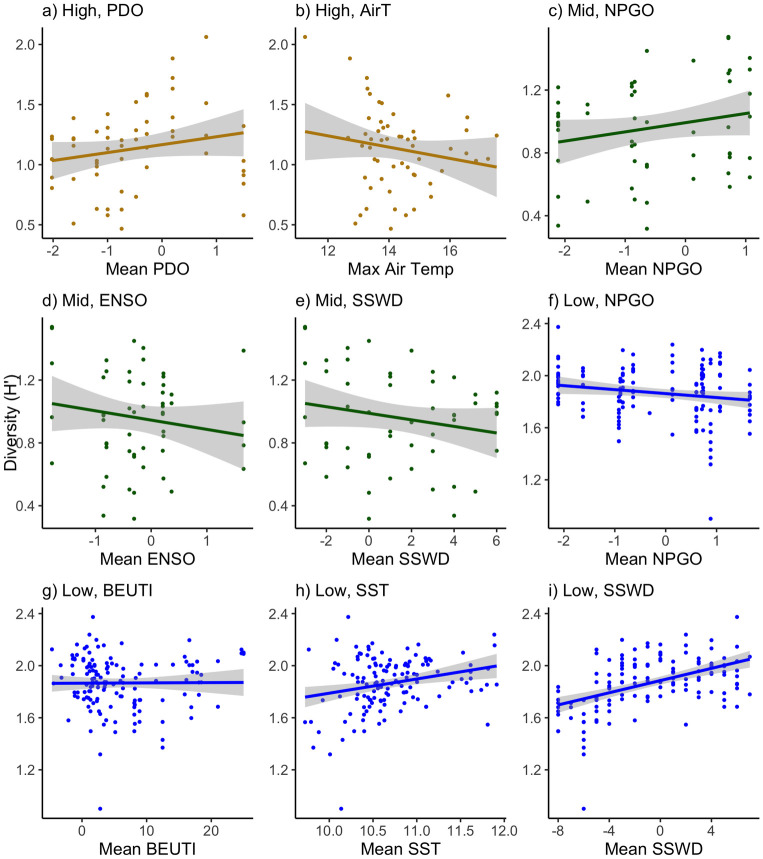
Linear correlations of taxon diversity (Shannon-Weiner index ± SE) with significant environmental drivers (identified by models in Table 4) in Oregon and northern California from 2006–2020. Zones included the a-b) high, c-e) mid, and f-i) low zones (yellow, green and blue, respectively). Environmental drivers included a) Pacific Decadal Oscillation (mean PDO), b) the maximum daily air temperature by site (Max Air Temp), c & f) North Pacific Gyre Oscillation (mean NPGO), d) Multivariate El Niño Southern Oscillation Index (mean ENSO), e & i) the years since the initial sea star wasting disease outbreak (Yrs since SSWD), g) Biologically Effective Upwelling Transport Index (mean BEUTI), and h) the mean daily water temperature by site (Mean SST).

Taxon diversity (H’) varied among sites, years, and their interaction in all three zones but most changes were small, accounting for little variance (S7 Fig in S3 Appendix; S4 Table in S1 Appendix). For example high zone diversity increased slightly with warmer PDO and cooler maximum air temperatures (R^2^ = 0.042 and 0.029, respectively; Fig 11a&11b; Table 5a). Mid zone diversity increased with windier NPGO and La Niña conditions and decreased with years since SSWD (R^2^ = 0.040, 0.029, & 0.006, respectively; Fig 11c–11e; Table 5b;). Low zone diversity increased with calmer NPGO conditions, increased upwelling, and warmer sea surface temperatures (R^2^ = 0.030, 0.001, & 0.065, respectively; Fig 11f–11h; Table 5c). The strongest trend was increased diversity with years since SSWD in the low zone (R^2^ = 0.229; Fig 11i, Table 5c).

**Table 5:** Linear model results testing the trends in taxon diversity (Shannon Index) with mean Pacific Decadal Oscillation index (mean PDO), mean North Pacific Gyre Oscillation index (mean NPGO), mean El Niño Southern Oscillation index (mean ENSO), mean and standard deviation of the Biologically Effective Upwelling Transport Index index (mean and sd BEUTI, respectively), mean daily sea surface temperature (Mean SST), mean daily maximum air temperature (Max Air Temp), and years since the sea star wasting disease epidemic (Yr Since SSWD). Model terms meeting the Bonferroni cutoff of P < 0.002 are in bold, and for these we subsequently tested the bivariate correlation of each significant term with diversity (bold indicates a bivariate correlation > 0.10).

Term	Chisq	Df	Pr(>Chisq)	Bivariate R^2^
**a) High zone**				
**Mean PDO**	10.21	1	**<0.001**	0.042
**Mean NPGO**	0.19	1	0.660	
**ENSO**	5.29	1	0.021	
**BEUTI**	6.15	1	0.013	
**SD BEUTI**	2.24	1	0.134	
**Max Air Temp**	17.49	1	**<0.001**	0.029
**Mean SST**	2.39	1	0.122	
**Yr Since SSWD**	2.71	1	0.099	
**b) Mid zone**				
**Mean PDO**	1.18	1	0.277	
**Mean NPGO**	87.94	1	**<0.001**	0.040
**ENSO**	38.28	1	**<0.001**	0.029
**BEUTI**	5.22	1	0.022	
**SD BEUTI**	0.06	1	0.805	
**Max Air Temp**	0.69	1	0.406	
**Mean SST**	9.02	1	0.003	
**Yr Since SSWD**	76.56	1	**<0.001**	0.006
**c) Low zone**				
**Mean PDO**	6.50	1	0.011	
**Mean NPGO**	17.51	1	**<0.001**	0.030
**ENSO**	4.01	1	0.045	
**BEUTI**	9.97	1	**0.002**	0.001
**SD BEUTI**	5.26	1	0.022	
**Max Air Temp**	0.16	1	0.688	
**Mean SST**	18.36	1	**<0.001**	0.065
**Yr Since SSWD**	68.03	1	**<0.001**	**0.229**

## Discussion

### Marked resistance of intertidal communities to oceanic variation

Our most striking finding was the consistency in rocky intertidal communities along the Oregon and Northern California coasts over the 14 years of study. We found little to no support for strong oceanic climate-driven fluctuations in community structure (Figs 7–9, Table 2), and only modest correlations between taxon abundance and climate fluctuations (Table 3). Analyses of rates of community change and change in diversity yielded similar results (Tables 3 and 4), suggesting that communities within the Oregon and northern California intertidal meta-ecosystem were resistance to environmental variation, at least during the 14 years of our study for the particular community parameters measured. Since several key ecological processes (e.g. growth, recruitment, resilience) have declined or become more variable with to climate change [11,12], this apparent resistance came as a surprise.

We expected intertidal communities to be sensitive to oceanic climate for several reasons. First, several studies have found evidence of range shifts or shifting dominance of northern/southern species in the intertidal over the past decades [67,68,98], and rocky intertidal communities are widely expected to be particularly sensitive to a changing climate [99]. Second, other studies in this system have detected sensitivity of ecological processes to ocean climate fluctuations like PDO, NPGO, ENSO upwelling, and temperature [20,54,62,64,100]. Third, there were multiple notable climate-related events during this time frame that we captured in our analysis (Figs 2 and 3). Most notably, there were several concurrent and interconnected shifts in 2014–2016 that included a severe marine heat wave (“the blob”), a strong El Niño event, and a shift to less windy NPGO and warmer PDO conditions [46,47,101]. While other studies have found changes in intertidal communities following these particular climate events [102], we did not. Fourth, our recent experiments at many of these same sites showed that recovery rates from annual experimental disturbance are declining and becoming more variable, suggesting declining resilience [11]. Additionally, several key ecological metrics (ecological subsidies, mussel performance, and sea star performance) are either diminishing or increasing in variability [12]. Below we discuss the apparent disconnect between ecological processes and community patterns, ask why this apparent resistance might exist, explore the subtle changes that we were able to detect in our study, and contemplate whether we can expect this resistance to continue in the future.

### The disconnect between varying ecological processes and emergent community structure

Our analysis suggests that as of 2020, the general structure of rocky intertidal communities on our shore appeared resistant to climate change. What is the underlying basis for the resistance of these communities to oceanic climate variation? We explore two ecological and possibilities: 1) time lags in responsiveness deriving from longevity of component species and 2) importance of top-down trophic forcing. First, many species in our system are long-lived and possess strong regenerative capabilities. For example, once they survive the extremely high mortality rates experienced as larvae and juveniles, many invertebrates in our system can live for years, decades, and even centuries. Some examples include sea anemones (*Anthopleura xanthogrammica* and *A*. *elegantissima)* that may live >100 years [103], sea urchins (*Strongylocentrotus purpuratus)* that likely live several decades [104], and mussels and large barnacles (*Mytilus californianus* and *Semibalanus cariosus)* that can live at least ~15 years [105,106]. Similarly, many of the dominant algae die back annually or when stressed, but can quickly regrow from their holdfasts [107–110]. So, while changes in climate mode or stress events can have sub-lethal physiological effects on these species [64,111–114], this may not translate into long-lasting changes in abundance. In addition, long-life spans of many component species mean that climate-sensitive processes like recruitment and growth may take years to translate into community change. Ultimately, long-life span and perennating capabilities suggest that sensitivity at the community level may lag behind the changes in oceanic climate and may not be detectable for years to come.

Second, climate-sensitive rates of recruitment and growth of individuals versus their survival in the face of top-down trophic forcing may influence the disconnect between ecological processes and community structure. For example, recruitment and/or growth rates of short-lived species of barnacles and mussels (*B*. *glandula*, *C*. *dalli*, and the mussel *M*. *trossulus*) at some of our sites (primarily sites on Cape Perpetua) can be extremely high [20,48,54,62,115]. However, while adult cover of these weedy species may spike as a result, these increases do not usually persist more than a few weeks to months due to high annual mortality from predation [116]. In fact, climate-driven high recruitment may simply transfer biomass to the top trophic levels, since these sites have high whelk and sea star densities [117,118]. Thus, predators may mute community sensitivity to climate fluctuations by transferring excess prey biomass to higher growth and reproductive output, as found for whelks by Navarrete et al. [116] and sea stars by Menge et al. [119].

### Shifts in intertidal communities with sea star wasting disease and climate fluctuations

Our analysis did detect a shift in rocky intertidal communities in relation to the years since sea star wasting disease, which caused sea star biomass to decline by 60–90% [9]. We found that SSWD accounted for 15.9% of variation in community structure in the mid zone (Table 2b) and was correlated to an increase in mussels (Fig 8, Table 3b), and a decrease in diversity (Fig 11e, Table 5b). These findings are consistent with the keystone predation hypothesis [73,74] that suggests *P*. *ochraceus* increases diversity by consuming mussels, thereby freeing space for other taxa in the intertidal zone thus increasing diversity. In the high zone, diversity showed an unexpected opposite trend with sea star wasting disease (Fig 11i, Table 5c). Upon further inspection, however, this seemed to be driven by low diversity estimates in the early part of the study (2006–2008) while no increase was evident since the outbreak in 2014. This suggests this trend is not related to the disease but instead may be due to low taxonomic resolution in the early years. The response to SSWD was not the primary focus of the present study but was included because it was a major event that was concurrent with the focal climate fluctuations. A more extensive investigation of this SSWD event will be reported elsewhere.

Averaging across sites and capes, mobile gastropods including limpets (*Lottia* species), herbivorous snails (primarily *Littorina* spp. and *Tegula funebralis*) and predatory snails (primarily *Nucella ostrina* and *N*. *canaliculata*) were quite variable over time (S4, S5 and S6 Figs in S3 Appendix). Some trends were notable (i.e. bivariate trends in Fig 8 showing an R^2^ of >0.10, in Table 3b), including increased limpets with windier NPGO in the high and mid zones, increased limpets with windier NPGO and higher upwelling variability in the high zone, and decreased predatory snails with warmer air temperatures in the mid zone. Limpets and whelks are relatively small and short-lived (e.g., 2–5 yr; [120–122]) so are likely susceptible to environmental stress. Thus, these species may be indicators of climate change, suggesting that the modest patterns noted above warrant further investigation.

We were surprised by the lack of responses of algal taxa to climate fluctuations, since they are primary producers that should conceivably be sensitive to variation in nutrients, temperature and environmental stress [123]. In particular, we expected kelps to be responsive to oceanic climate since others have noted negative responses to El Niño and temperature [64,124]. Further, subtidal bull kelp (*Nereocystis luetkeana*) populations in Oregon and northern California collapsed just adjacent to several of our sites due to a combination of warm water and sea urchin overgrazing [75,125]. However, despite the clear effects of climate on nearby subtidal kelps, the intertidal kelps (primarily *Hedophyllum sessile*) in our study were markedly consistent. This may be linked to their ability to to perennate and regrow from their holdfasts each year [108] unlike the nearby subtidal canopy-forming kelps [126]. The only notable trends in algal/plant taxa were an increase in the mid-to high zone species *Endocladia muricata* in the mid zone only during years of high air temperature (Fig 8, Table 3b), which could indicate a seaward shift in hot years. Surfgrass (*Phyllospadix scouleri* and *P*. *serrulatus*) abundance also increased with upwelling intermittency in the low zone (Fig 9, Table 3c), which may be linked to higher nutrients in the water during these years [16].

### Limited inferences for long-term stability and the potential for imminent change

It is possible that the 14-year duration of our study may have been too short to detect climate-induced change, especially in relation to the longer-period climate cycles like PDO and NPGO (20–30 year and 7–15 year cycles, respectively). For example, the declines in mussel cover and increases in algal cover referred to earlier occurred over 20 years [54], and a ~30 year study recently found that west coast intertidal communities were shifting northward at about 3.5–5 km/yr [98]. While it is certainly true that further decades of study are necessary before we can make concrete determinations, several other studies performed at similar decade-long timescales have detected sensitivity of ecological processes to oceanic climate fluctuations in this system during this period [20,36,54,62]. This suggests our approach is robust, and if strong and immediate effects of these cycles were occurring, we should have been able to detect them.

On the other hand, it is likely that there are responses to climate change occurring in this system that we were not able to detect with the indicators of annual community structure, functional group abundance, and diversity we measured here. For example, our plots were not fixed in space, so we were unable to detect small changes in cover of any given species over time. We also did not focus on rarer species that may be responding to climate change. Also, unlike latitudinally-focused studies that have detected range shifts over dozens or hundreds of intertidal sites [67,98,99] our 14-site study was well within the range edges of most of our functional groups, so we cannot adequately investigate range shifts here. Finally, unlike others who have detected sensitivity to seasonal environmental changes in upwelling or to events like heat waves and storms [34,65,102,127], our annual surveys were unable to resolve any intra-annual response.

Overall, the detected resistance of intertidal communities to climate change is only relevant within the environmental regime encompassed by the 14-year time frame of our study. Because intertidal communities are expected to be particularly prone to a changing climate and because several studies have already uncovered sensitivity of intertidal species and communities to climate change [reviewed by 98], readers should interpret our results with caution. For example, as climate change intensifies these communities could reach tipping points and undergo sudden near-future phase shifts in response to extreme events, species invasions, sea level rise, or disease outbreaks [17,42,128,129]. Some evidence suggests that ecological effects of climate change may manifest as rapid ecological shifts after extreme events [130]. Examples include drought-driven fires in forests or marine heatwave driven bleaching events in coral reefs. A relevant case was the Pacific Northwest ‘heat dome’ event in late June 2021 that caused several days of record heat with 50°C temperatures recorded on intertidal surfaces in the Salish Sea and killed around 70% mussels and barnacles [131]. In this case, however, Oregon’s communities were spared because in June the daily lower low tides occur in early morning, i.e., prior to the hottest mid-day hours. But more incremental climate change may also eventually cause shifts. With the previously discussed climate-related changes in invertebrate recruitment, growth, and reproduction, it seems probable that as more species in these systems respond to shifting climate at population or sub-organismal levels, the apparent inertia of entire communities to climate-related change may eventually be overcome. Indeed, the very clear spatial differences we and others [50,53,132] observed geographically, and the shifts documented by Raimondi et al. [98] suggest that cumulative differences in oceanic climate will eventually cause community shifts. Further, it is widely expected that sea level rise will have profound effects on rocky intertidal communities [133,134]. Ultimately, further research is needed to capture community responses to both within-year drivers like upwelling and heat waves and responses to among-decade drivers like ENSO, NPGO and PDO.

## Conclusion

The intertidal communities in this meta-ecosystem appeared resistant to multiple oceanic climate drivers, at least within the scope of environmental variation observed in our 14-year timescale. This apparent resistance to oceanic climate is especially notable because previous research indicates that multiple ecological processes are sensitive to one or more of these climate drivers, and that this system has recently shown signs of diminishing resilience to disturbance [11,12]. Thus, there appears to be a disconnect between the sensitivity of ecological processes to climate variability and the emergent structure of these ecological communities. We suggest that the longevity of many taxa or the importance of top-down forcing might be responsible for this disconnect. While these results are promising for the fate of these communities in the era of rapid global change, we are cautious with our optimism. It is possible that our study was too short for the effects of climate fluctuations to manifest, that our indicators were too coarse, or that response to changing climate is non-linear, and that we have not yet observed the conditions that would elicit a “tipping point”. Finally, catastrophic events like extreme weather, marine heat waves, and disease outbreaks are increasing with climate change, and these could have sudden and drastic effects on these communities. Regardless, the rocky intertidal communities of Oregon and Northern California appear to have been broadly resistant to oceanic climate fluctuations over the past 14 years.

## Supporting information

S1 Appendix Supplemental tables. (DOCX)

S2 Appendix Background on ocean climate indices. (DOCX)

S3 Appendix Supplemental figures. (DOCX)
