# Supplementary material for: Resistance of rocky intertidal communities to oceanic climate fluctuations
Source: PLoS One. 2024 May 29;19(5):e0297697. doi: 10.1371/journal.pone.0297697 (PMC11135789; doi:10.1371/journal.pone.0297697)
Supplement: S1 Appendix — (DOCX) [file pone.0297697.s001.docx]

**Appendix S1: Supplementary Tables**

**Resistance of rocky intertidal communities to oceanic climate fluctuations**

Sarah A. Gravem, Brittany Poirson, Jonathan Robinson, and Bruce A. Menge

Department of Integrative Biology, Oregon State University, Corvallis, OR 97331

# Table S1. List of sites surveyed in the study

| **Site** | **Site Code** | **Cape** | **Latitude** | **Longitude** | **Zones** |
| --- | --- | --- | --- | --- | --- |
| Fogarty Creek | FC | Foulweather | 44.83864 | -124.05875 | All |
| Boiler Bay | BB | Foulweather | 44.83033 | -124.06078 | All |
| Yachats Beach | YB | Perpetua | 44.31140 | -124.10860 | All |
| Strawberry Hill | SH | Perpetua | 44.24923 | -124.11543 | All |
| Tokatee Klootchman | TK | Perpetua | 44.20368 | -124.11704 | Low |
| Cape Blanco North | CBN | Blanco | 42.83969 | -124.56512 | All |
| Cape Blanco South | CBS | Blanco | 42.83440 | -124.56530 | Low |
| Port Orford Heads | POH | Blanco | 42.74394 | -124.51415 | Low |
| Rocky Point | RP | Blanco | 42.71777 | -124.46637 | All |
| Cape Mendocino | CMEN | Mendocino | 40.34100 | -124.36317 | Low |
| Cape Mendocino | CMES | Mendocino | 40.34122 | -124.36326 | Low |

# Table S2. List of species or taxa quantified in the field and used for taxon biodiversity. Functional groupings indicate assignments used for community analyses.

| **Species** | **Functional Group** | **Species** | **Functional Group** |
| --- | --- | --- | --- |
| Bare space | Bare Space | *Phyllospadix scouleri* | Surf grass |
| Gravel/Cobble | Bare Space | *Phyllospadix serrulatus* | Surf grass |
| Sand | Bare Space | Crustose coralline algae | Coralline algae crusts (CCA) |
| Diatoms | Diatoms | *Bossiella plumosa* | Articulated coralline algae |
| brown filamentous | Diatoms | *Calliarthron tuberculosum* | Articulated coralline algae |
| Brown/Red Crust | Algal crusts | corralline holdfasts | Articulated coralline algae |
| *Fucus distichus* | Fucoid algae | *Corallina* spp. | Articulated coralline algae |
| *Pelvetiopsis limitata* | Fucoid algae | *Constantinea simplex* | Constantinea |
| *Hedophyllum sessile* | Hedophyllum | *Cryptopleura ruprechtiana* | Cryptopleura & Hymenena |
| *Postelsia palmaeformis* | Postelsia | *Hymenena flabelligera* | Cryptopleura & Hymenena |
| *Alaria marginata* | Kelps | *Dilsea californica* | Dilsea |
| *Costaria costata* | Kelps | *Egregia menziesii* | Egregia |
| *Desmarestia* spp. | Kelps | *Endocladia muricata* | Endocladia |
| *Laminaria setchellii* | Kelps | *Mastocarpus jardinii* | Mastocarpus |
| *Laminaria sinclairii* | Kelps | *Mastocarpus papillatus* | Mastocarpus |
| *Lessoniopsis littoralis* | Kelps | *Mazzaella flaccida* | Mazzaella blades |
| *Nereocystis luetkeana* | Kelps | *Mazzaella linearus* | Mazzaella blades |
| *Analipus japonicus* | Brown understory algae | *Mazzaella splendens* | Mazzaella blades |
| Brown Holdfast | Brown understory algae | *Neorhodomela larix* | Odonthalia and Neorhodomela |
| *Colpomenia spp. /Leathesia difformis* | Brown understory algae | *Odonthalia floccosa* | Odonthalia & Neorhodomela |
| *Ectocarpus* spp. | Brown understory algae | *Odonthalia oregona* | Odonthalia & Neorhodomela |
| *Scytosiphon* spp. | Brown understory algae | *Pyropia* spp. | Pyropia |
| *Soranthera ulvoidea* | Brown understory algae | *Cryptosiphonia woodii* | Fine branching reds |
| Unidentified foliose brown | Brown understory algae | *Microcladia borealis* | Fine branching reds |
| *Ulva* spp. | Ulva | *Plocamium spp.* | Fine branching reds |
| *Acrosiphonia coalita* | Green algae | *Polysiphonia* spp. | Fine branching reds |
| *Bryopsis* spp. | Green algae | red filamentous | Fine branching reds |
| *Chaetomorpha* spp. | Green algae | *Chondracanthus exasperatus* | Red blades |
| *Cladophora* spp. | Green algae | *Erythrophyllum delesserioides* | Red blades |
| *Codium* spp. | Green algae | *Gastroclonium subarticulatum* | Red blades |
| *Derbesia marina* | Green algae | *Gelidium spp.* | Red blades |
| Filamentous green algae | Green algae | *Gigartina canaliculata* | Red blades |
| Green Holdfast | Green algae | *Gymnogongrus griffithsiae* | Red blades |

| **Taxon** | **Functional Group** | **Taxon** | **Functional Group** |
| --- | --- | --- | --- |
| *Halosaccion glandiforme* | Red blades | Colonial tunicate | Tunicates |
| *Mazzaella affinis* | Red blades | Stalked tunicate | Tunicates |
| *Opuntiella californica* | Red blades | *Strongylocentrotus purpuratus* | Sea urchins |
| *Palmaria spp.* | Red blades | Sea Cucumber | Sea cucumbers |
| *Phaeostrophion irregulare* | Red blades | *Henricia* spp. | Other Sea stars |
| *Prionitis lanceolata* | Red blades | *Leptasterias* spp. | Predatory sea stars |
| red blade | Red blades | *Pisaster ochraceus* | Predatory sea stars |
| *Rhodymenia pacifica* | Red blades | *Lacuna* spp. | Herbivorous snails |
| *Schizymenia pacifica* | Red blades | *Littorina* spp. | Herbivorous snails |
| *Ahnfeltiopsis linearis* | Red turfs | *Tegula* spp. | Herbivorous snails |
| *Ahnfeltia fastigiata* | Red turfs | *Cryptochiton stelleri* | Chitons |
| Branched red algae | Red turfs | *Katharina tunicata* | Chitons |
| *Callithamnion pikeanum* | Red turfs | *Lepidozona* spp. | Chitons |
| *Callophyllis* spp. | Red turfs | *Mopalia* spp. | Chitons |
| *Ceramium* spp. | Red turfs | *Tonicella lineata* | Chitons |
| *Farlowia mollis* | Red turfs | Chiton | Chitons |
| Foliose red algae | Red turfs | *Acmaea mitra* | Limpets |
| *Gloiopeltis furcata* | Red turfs | *Lottia digitalis* | Limpets |
| *Mazzaella parksii* | Red turfs | *Lottia pelta* | Limpets |
| *Osmundea spectabilis* | Red turfs | *Lottia strigatella* | Limpets |
| *Ptilota filicina* | Red turfs | *Lottia* spp. | Limpets |
| Red Holdfast | Red turfs | *Anisodoris nobilis* | Nudibranchs |
| *Mytilus californianus* | Mussels | *Archidoris spp.* | Nudibranchs |
| *Mytilus trossulus* | Mussels | *Onchidoris bilamellata* | Nudibranchs |
| *Balanus glandula* | Balanus | *Amphissa columbiana* | Predatory snails |
| *Balanus spp.* | Balanus | *Nucella canaliculata* | Predatory snails |
| *Chthamalus dalli* | Chthamalus | *Nucella ostrina* | Predatory snails |
| *Balanus nubilus* | Large barnacles | whelk | Predatory snails |
| *Semibalanus cariosus* | Large barnacles | *Cancer productus* | Crabs |
| *Pollicipes polymerus* | Gooseneck barnacles | Crab | Crabs |
| *Anthopleura elegantissima* | Anemones | *Hemigrapsus* spp. | Crabs |
| *Anthopleura xanthogrammica* | Anemones | Hermit Crab | Crabs |
| *Epiactis prolifera* | Anemones | *Pagurus hirsutiusculus* | Crabs |
| Unidentified Anemone | Anemones | *Pugettia producta* | Crabs |
| Bryozoans | Bryozoans | Amphipod | Small crustaceans |
| Hydrozoan | Hydrozoans | Isopod | Small crustaceans |
| Sponge | Sponges | Nemertean | Worms |
| Tube Worms | Tube worms | Sipunculid | Worms |
|  |  | Worm | Worms |

# Table S3. Results of 3 PERMANOVA and SIMPER analyses. a-c) PERMANOVAs testing the effects of capes, sites, years, and their interactions on intertidal community dissimilarity among the a) high, b) mid, and c) low zones in Oregon and northern California from 2006-2021. d-f) Results of SIMPER analyses testing which functional groups were most associated with within-cape similarity for the d), high e), mid and f) low zone. Only functional groups contributing most strongly to within-cape similarity are listed. Communities were analyzed at the quadrat level in the mid and high zones (0.5 x 0.5m) and the transect level in the low zone (the average of ~10 0.5 x 0.5m quadrats).

1. PERMANOVA High zone

| **Source** | **df** | **SS** | **MS** | **Pseudo-F** | **P** | **Estimate** | **Sq.root** | **% Explained** |
| --- | --- | --- | --- | --- | --- | --- | --- | --- |
| **Cape** | 2 | 89,228 | 44,614 | 1.599 | 0.083 | 99 | 9.9 | 7.2 |
| **Year** | 10 | 101,140 | 10,114 | 2.900 | 0.001 | 129 | 11.4 | 9.4 |
| **Site[Cape]** | 3 | 75,324 | 25,108 | 6.852 | 0.001 | 248 | 15.7 | 18.0 |
| **Year*Cape** | 18 | 60,186 | 3,344 | 0.912 | 0.639 | -18 | -4.2 | -1.3 |
| **Year*Site[Cape]** | 23 | 84,273 | 3,664 | 5.975 | 0.001 | 306 | 17.5 | 22.2 |
| **Residual** | 517 | 317,020 | 613 |  |  | 613 | 24.8 | 44.5 |
| **Total** | 573 | 742,780 |  |  |  | 1378 |  |  |

1. PERMANOVA Mid zone

| **Source** | **df** | **SS** | **MS** | **Pseudo-F** | **P** | **Estimate** | **Sq.root** | **% explained** |
| --- | --- | --- | --- | --- | --- | --- | --- | --- |
| **Cape** | 2 | 85,103 | 42,551 | 2.170 | 0.008 | 138 | 11.7 | 10.8 |
| **Year** | 9 | 88,405 | 9,823 | 3.182 | 0.001 | 124 | 11.1 | 9.7 |
| **Site[Cape]** | 3 | 51,929 | 17,310 | 5.340 | 0.001 | 163 | 12.8 | 12.7 |
| **Year*Cape** | 17 | 51,427 | 3,025 | 0.933 | 0.634 | -12 | -3.4 | -0.9 |
| **Year*Site[Cape]** | 23 | 73,686 | 3,204 | 5.281 | 0.001 | 261 | 16.1 | 20.4 |
| **Residual** | 500 | 303,330 | 607 |  |  | 607 | 24.6 | 47.4 |
| **Total** | 554 | 663,550 |  |  |  | 1279.93 |  |  |

1. PERMANOVA Low zone

| **Source** | **df** | **SS** | **MS** | **Pseudo-F** | **P** | **Estimate** | **Sq.root** | **% Explained** |
| --- | --- | --- | --- | --- | --- | --- | --- | --- |
| **Cape** | 3 | 96,149 | 32,050 | 3.312 | 0.001 | 274 | 16.6 | 22.7 |
| **Year** | 14 | 50,763 | 3,626 | 4.009 | 0.001 | 120 | 11.0 | 10.0 |
| **Site[Cape]** | 7 | 56,674 | 8,096 | 12.387 | 0.001 | 252 | 15.9 | 20.9 |
| **Year*Cape** | 38 | 32,556 | 857 | 1.325 | 0.001 | 35 | 5.9 | 2.9 |
| **Year*Site[Cape]** | 76 | 48,876 | 643 | 1.444 | 0.001 | 79 | 8.9 | 6.6 |
| **Residual** | 210 | 93,521 | 445 |  |  | 445.34 | 21.1 | 36.9 |
| **Total** | 348 | 382,690 |  |  |  | 1205.83 |  |  |

1. SIMPER High Zone

| **Taxon** | **Av.Abund** | **Av.Sim** | **Sim/SD** | **Contrib%** | **Cum.%** |
| --- | --- | --- | --- | --- | --- |
| **Cape Foulweather** | | | | | |
| Bare Space | 3.45 | 14.17 | 2.02 | 27.57 | 27.57 |
| Herbivorous snails | 3.3 | 7.43 | 0.78 | 14.45 | 42.01 |
| Balanus | 2.27 | 6.48 | 1.11 | 12.61 | 54.62 |
| Fucoid algae | 2.08 | 6.19 | 1.12 | 12.05 | 66.67 |
| Limpets | 2.27 | 4.95 | 0.79 | 9.64 | 76.31 |
| Chthamalus | 1.6 | 4.07 | 0.92 | 7.92 | 84.23 |
| Mastocarpus | 1.04 | 2.61 | 0.95 | 5.07 | 89.31 |
| Mussels | 1.19 | 1.98 | 0.56 | 3.85 | 93.16 |
| Green algae | 0.85 | 1.15 | 0.4 | 2.24 | 95.4 |
| Endocladia | 0.55 | 0.69 | 0.42 | 1.35 | 96.75 |
| **Cape Perpetua** | | | | | |
| Bare Space | 3.16 | 14.24 | 2.15 | 28.19 | 28.19 |
| Balanus | 2.88 | 10.72 | 1.17 | 21.21 | 49.4 |
| Herbivorous snails | 3.6 | 9.22 | 0.78 | 18.24 | 67.64 |
| Fucoid algae | 1.71 | 4.52 | 0.71 | 8.95 | 76.59 |
| Mussels | 1.26 | 2.98 | 0.73 | 5.9 | 82.49 |
| Limpets | 1.55 | 2.94 | 0.59 | 5.83 | 88.32 |
| Chthamalus | 0.77 | 1.58 | 0.6 | 3.13 | 91.44 |
| Mastocarpus | 0.7 | 1.23 | 0.5 | 2.43 | 93.88 |
| Endocladia | 0.59 | 0.8 | 0.37 | 1.58 | 95.46 |
| Large barnacles | 0.65 | 0.65 | 0.22 | 1.29 | 96.74 |
| **Cape Blanco** | | | | | |
| Bare Space | 4.09 | 22.41 | 3 | 36.06 | 36.06 |
| Limpets | 3.88 | 14.68 | 1.79 | 23.62 | 59.68 |
| Balanus | 2.59 | 10.3 | 1.67 | 16.57 | 76.25 |
| Herbivorous snails | 2.71 | 5.97 | 0.68 | 9.61 | 85.85 |
| Chthamalus | 1.08 | 3.21 | 1.04 | 5.16 | 91.02 |
| Algal crusts | 0.91 | 2.41 | 0.88 | 3.88 | 94.9 |
| Mussels | 0.56 | 1.09 | 0.6 | 1.75 | 96.65 |
| Mastocarpus | 0.31 | 0.56 | 0.39 | 0.9 | 97.55 |
| Endocladia | 0.43 | 0.54 | 0.36 | 0.87 | 98.42 |
| Fucoid algae | 0.49 | 0.48 | 0.25 | 0.77 | 99.19 |

1. SIMPER Mid Zone

| **Taxon** | **Av.Abund** | **Av.Sim** | **Sim/SD** | **Contrib%** | **Cum.%** |
| --- | --- | --- | --- | --- | --- |
| **Cape Foulweather** | | | | | |
| Mussels | 3.85 | 22.06 | 1.92 | 43.48 | 43.48 |
| Limpets | 3.27 | 12.9 | 1.16 | 25.42 | 68.9 |
| Gooseneck barnacles | 1.22 | 2.99 | 0.61 | 5.9 | 74.8 |
| Bare Space | 1.28 | 2.98 | 0.7 | 5.88 | 80.68 |
| Predatory snails | 1.08 | 2.83 | 0.7 | 5.57 | 86.25 |
| Large barnacles | 1.18 | 2.15 | 0.51 | 4.24 | 90.5 |
| Articulated coralline algae | 1.03 | 1.68 | 0.42 | 3.32 | 93.81 |
| Predatory sea stars | 0.53 | 0.88 | 0.4 | 1.74 | 95.55 |
| Balanus | 0.6 | 0.57 | 0.25 | 1.12 | 96.67 |
| Coralline algae crusts | 0.43 | 0.4 | 0.25 | 0.79 | 97.47 |
| **Cape Perpetua** | | | | | |
| Mussels | 4.14 | 24.9 | 2.35 | 42.45 | 42.45 |
| Limpets | 3.33 | 12.54 | 1.25 | 21.38 | 63.83 |
| Predatory snails | 2.55 | 9.96 | 1.33 | 16.98 | 80.8 |
| Bare Space | 1.37 | 3.72 | 0.81 | 6.34 | 87.15 |
| Large barnacles | 1.23 | 2.68 | 0.59 | 4.57 | 91.72 |
| Gooseneck barnacles | 1.03 | 2.62 | 0.53 | 4.47 | 96.19 |
| Balanus | 0.48 | 0.5 | 0.29 | 0.86 | 97.05 |
| Herbivorous snails | 0.7 | 0.47 | 0.19 | 0.8 | 97.84 |
| Anemones | 0.36 | 0.46 | 0.34 | 0.79 | 98.63 |
| Chthamalus | 0.37 | 0.42 | 0.32 | 0.71 | 99.34 |
| **Cape Blanco** | | | | | |
| Mussels | 4.34 | 37.51 | 2.54 | 58.9 | 58.9 |
| Limpets | 3.46 | 20.3 | 1.73 | 31.88 | 90.78 |
| Predatory snails | 0.66 | 1.8 | 0.48 | 2.83 | 93.61 |
| Endocladia | 0.68 | 1.52 | 0.34 | 2.39 | 96.01 |
| Bare Space | 0.81 | 1.3 | 0.32 | 2.03 | 98.04 |
| Large barnacles | 0.45 | 0.54 | 0.24 | 0.85 | 98.89 |
| Gooseneck barnacles | 0.17 | 0.21 | 0.2 | 0.33 | 99.22 |
| Balanus | 0.24 | 0.19 | 0.2 | 0.31 | 99.52 |
| Chthamalus | 0.18 | 0.14 | 0.17 | 0.21 | 99.73 |
| Pyropia | 0.13 | 0.07 | 0.1 | 0.1 | 99.84 |

1. SIMPER Low zone

| **Taxon** | **Av.Abund** | **Av.Sim** | **Sim/SD** | **Contrib%** | **Cum.%** |
| --- | --- | --- | --- | --- | --- |
| **Cape Foulweather** | | | | | |
| Articulated coralline algae | 2.53 | 8.41 | 4.57 | 12.78 | 12.78 |
| Bare Space | 2.4 | 7.5 | 3.01 | 11.4 | 24.17 |
| Coralline algae crusts | 2.21 | 7.16 | 4.12 | 10.87 | 35.04 |
| Hedophyllum | 1.99 | 5.09 | 1.37 | 7.74 | 42.78 |
| Cryptopleura and Hymenena | 1.5 | 4.04 | 1.82 | 6.14 | 48.93 |
| Surf grass | 1.58 | 3.91 | 1.4 | 5.94 | 54.87 |
| Dilsea | 1.31 | 3.52 | 1.7 | 5.35 | 60.22 |
| Mazzaella blades | 1.15 | 3.08 | 1.87 | 4.68 | 64.89 |
| Fine branching reds | 1.17 | 2.88 | 1.47 | 4.38 | 69.27 |
| Sea urchins | 1.12 | 2.82 | 1.66 | 4.29 | 73.56 |
| **Cape Perpetua** | | | | | |
| Bare Space | 2.89 | 10.58 | 3.3 | 17.79 | 17.79 |
| Articulated coralline algae | 2.37 | 8.33 | 2.84 | 14 | 31.8 |
| Anemones | 1.67 | 5.95 | 3.25 | 10 | 41.8 |
| Limpets | 2 | 4.81 | 1.2 | 8.09 | 49.89 |
| Predatory sea stars | 1.1 | 3.01 | 1.51 | 5.06 | 54.95 |
| Gooseneck barnacles | 1.42 | 2.97 | 0.74 | 4.99 | 59.93 |
| Dilsea | 1.03 | 2.88 | 1.4 | 4.84 | 64.78 |
| Coralline algae crusts | 0.98 | 2.48 | 1.17 | 4.18 | 68.95 |
| Chthamalus | 0.92 | 2.13 | 1.09 | 3.58 | 72.54 |
| Fine branching reds | 0.94 | 2.1 | 1 | 3.53 | 76.06 |
| **Cape Blanco** | | | | | |
| Bare Space | 3.06 | 11.11 | 3.85 | 19.94 | 19.94 |
| Articulated coralline algae | 1.98 | 5.98 | 1.8 | 10.74 | 30.68 |
| Limpets | 1.72 | 4.11 | 1.26 | 7.38 | 38.06 |
| Coralline algae crusts | 1.35 | 3.67 | 1.67 | 6.59 | 44.65 |
| Algal crusts | 1.26 | 3.61 | 1.93 | 6.48 | 51.13 |
| Mazzaella blades | 1.22 | 3.25 | 1.33 | 5.83 | 56.96 |
| Fine branching reds | 1.14 | 3.2 | 1.73 | 5.75 | 62.71 |
| Surf grass | 1.29 | 3.13 | 1.09 | 5.62 | 68.33 |
| Hedophyllum | 1.18 | 2.62 | 0.93 | 4.71 | 73.04 |
| Cryptopleura and Hymenena | 0.96 | 1.72 | 0.73 | 3.09 | 76.13 |
| **Cape Mendocino** | | | | | |
| Bare Space | 3.06 | 11.11 | 3.85 | 19.94 | 19.94 |
| Articulated coralline algae | 1.98 | 5.98 | 1.8 | 10.74 | 30.68 |
| Limpets | 1.72 | 4.11 | 1.26 | 7.38 | 38.06 |
| Coralline algae crusts | 1.35 | 3.67 | 1.67 | 6.59 | 44.65 |
| Algal crusts | 1.26 | 3.61 | 1.93 | 6.48 | 51.13 |
| Mazzaella blades | 1.22 | 3.25 | 1.33 | 5.83 | 56.96 |
| Fine branching reds | 1.14 | 3.2 | 1.73 | 5.75 | 62.71 |
| Surf grass | 1.29 | 3.13 | 1.09 | 5.62 | 68.33 |
| Hedophyllum | 1.18 | 2.62 | 0.93 | 4.71 | 73.04 |
| Cryptopleura and Hymenena | 0.96 | 1.72 | 0.73 | 3.09 | 76.13 |

# Table S4. Linear model results testing the trends in taxon biodiversity (Shannon-Weiner Index) among sites and years in each zone

| **Term** | **Df** | **SumSq** | **Mean Sq** | **F** | **Pr(>F)** |
| --- | --- | --- | --- | --- | --- |
| **High zone** | | | | | |
| **Year** | 9 | 24.63 | 2.74 | 24.19 | <0.001 |
| **Site** | 5 | 10.40 | 2.08 | 18.39 | <0.001 |
| **Year * Site** | 41 | 27.17 | 0.66 | 5.86 | <0.001 |
| **Residuals** | 503 | 56.91 | 0.11 |  |  |
| **Mid zone** | | | | | |
| **Year** | 9 | 9.87 | 1.10 | 9.27 | <0.001 |
| **Site** | 5 | 27.96 | 5.59 | 47.26 | <0.001 |
| **Year * Site** | 40 | 14.77 | 0.37 | 3.12 | <0.001 |
| **Residuals** | 500 | 59.17 | 0.12 |  |  |
| **Low zone** | | | | | |
| **Year** | 12 | 42.79 | 3.57 | 31.90 | <0.001 |
| **Site** | 8 | 16.00 | 2.00 | 17.89 | <0.001 |
| **Year * Site** | 96 | 46.05 | 0.48 | 4.29 | <0.001 |
| **Residuals** | 2831 | 316.49 | 0.11 |  |  |
