# Supplementary material for: Resistance of rocky intertidal communities to oceanic climate fluctuations
Source: PLoS One. 2024 May 29;19(5):e0297697. doi: 10.1371/journal.pone.0297697 (PMC11135789; doi:10.1371/journal.pone.0297697)
Supplement: S2 Appendix — (DOCX) [file pone.0297697.s002.docx]

**Appendix S2: Background on Ocean Climate Indices**

**Resistance of rocky intertidal communities to oceanic climate fluctuations**

Sarah A. Gravem, Brittany Poirson, Jonathan Robinson, and Bruce A. Menge

Department of Integrative Biology, Oregon State University, Corvallis, OR 97331

The periodicity of each climate index we investigated differs, varying from 20-30 years for PDO, 7–15 years for NPGO, 3–7 years for ENSO, and inter- and intra-annually for upwelling. PDO is a reflection of cycles in sea surface temperature over the North Pacific, with positive (negative) values indicating warmer (cooler) years (Mantua et al. 1997). NPGO is orthogonal to the PDO index and positive values indicate windier conditions and typically periods of high chlorophyll concentration along our coastline (Di Lorenzo et al. 2008, Menge et al. 2009). PDO is more influential above the 38^o^N parallel, which includes the latitudes of our sites, and NPGO is more influential southward (Di Lorenzo et al. 2008). ENSO is driven by changes in pressure, air and water temperature, wind, and cloudiness over the tropical Pacific (Wolter and Timlin 2011). Warm phases above an index value of 1.0 are El Niño years and are associated with reduced productivity compared to the more productive and cooler La Niña years (Chavez et al. 2003, Peterson and Schwing 2003). El Niño’s influence should be stronger at southern sites closer to the sub-tropical origin of the warmer waters. Upwelling is a result of alongshore wind stress (Bakun 1990) that pulls deep, nutrient-rich water up to the surface (Huyer 1983). Positive values indicate upwelling, and negative values indicate downwelling. Upwelling is strongest in the spring and work in our lab has shown that variability or intermittency in upwelling can be a strong drive of onshore dynamics because intermittently strong upwelling (typical of central Oregon sites) provides both enough nutrients and enough wind relaxation for phytoplankton blooms to develop nearshore, while persistently strong upwelling winds (typical of S. Oregon and N. California sites) transports the blooms offshore (Menge and Menge 2013, Menge et al. 2015).

Bakun, A. 1990. Global climate change and intensification of coastal ocean upwelling. Science 247:198–201.

Chavez, F. P., J. Ryan, S. E. Lluch-Cota, and C. M. Ñiquen. 2003. From anchovies to sardines and back: Multidecadal change in the Pacific Ocean. Science 299:217–221.

Huyer, A. 1983. Coastal upwelling in the California current system. Progress in Oceanography 12:259–284.

Di Lorenzo, E., N. Schneider, K. M. Cobb, P. J. S. Franks, K. Chhak, A. J. Miller, J. C. McWilliams, S. J. Bograd, H. Arango, E. Curchitser, T. M. Powell, and P. Rivière. 2008. North Pacific Gyre Oscillation links ocean climate and ecosystem change. Geophysical Research Letters 35:1–6.

Mantua, N. J., S. R. Hare, Y. Zhang, J. M. Wallace, and R. C. Francis. 1997. A Pacific Interdecadal Climate Oscillation with impacts on salmon production. Bulletin of the American Meteorological Society 78:1069–1079.

Menge, B. A., F. Chan, K. J. Nielsen, E. Di Lorenzo, and J. Lubchenco. 2009. Climatic variation alters supply-side ecology: impact of climate patterns on phytoplankton and mussel recruitment. Ecological Monographs 79:379–395.

Menge, B. A., T. C. Gouhier, S. D. Hacker, F. Chan, and K. J. Nielsen. 2015. Are meta-ecosystems organized hierarchically? A model and test in rocky intertidal habitats. Ecological Monographs 85:213–233.

Menge, B. A., and D. N. L. Menge. 2013. Dynamics of coastal meta-ecosystems: the intermittent upwelling hypothesis and a test in rocky intertidal regions. Ecological Monographs 83:283–310.

Peterson, W. T., and F. B. Schwing. 2003. A new climate regime in northeast Pacific ecosystems. Geophysical Research Letters 30:1–4.

Wolter, K., and M. S. Timlin. 2011. El Niño/Southern Oscillation behaviour since 1871 as diagnosed in an extended multivariate ENSO index (MEI.ext). International Journal of Climatology 31:1074–1087.
