# Supplementary material for: Resistance of rocky intertidal communities to oceanic climate fluctuations
Source: PLoS One. 2024 May 29;19(5):e0297697. doi: 10.1371/journal.pone.0297697 (PMC11135789; doi:10.1371/journal.pone.0297697)
Supplement: S3 Appendix — (DOCX) [file pone.0297697.s003.docx]

**Appendix S3. Supplementary Figures**

**Resistance of rocky intertidal communities to oceanic climate fluctuations**

Sarah A. Gravem, Brittany Poirson, Jonathan Robinson, and Bruce A. Menge

Department of Integrative Biology, Oregon State University, Corvallis, OR 97331


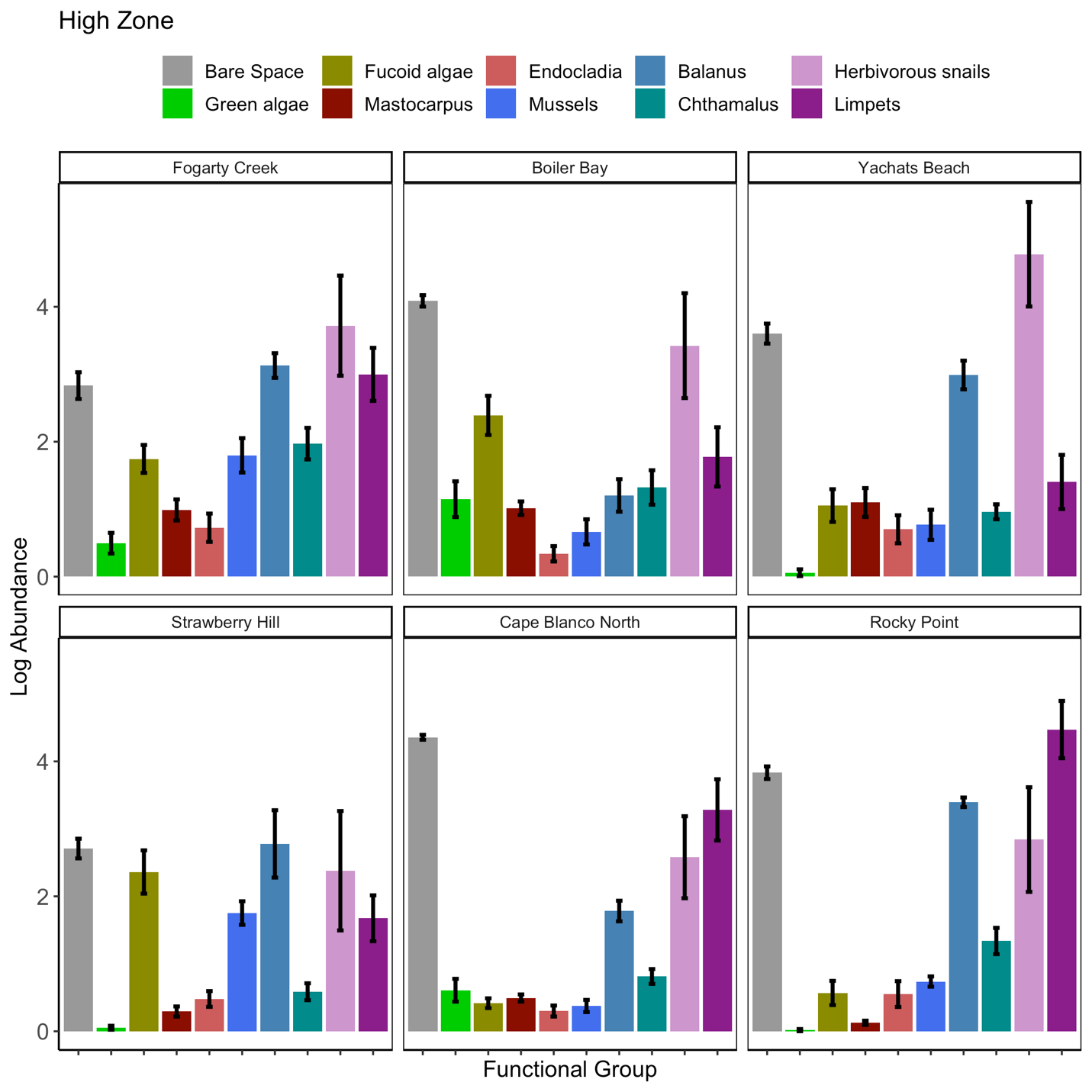


# **Figure S1.** Average (± SE) functional group abundances in the high intertidal zone among sites in Oregon and northern California from 2006-2020. Colors correspond to functional groups, with bare space in gray, green algae/plants in greens, brown algae in yellows, red algae in reds and pinks, sessile invertebrates in blues, and mobile invertebrates in purples.


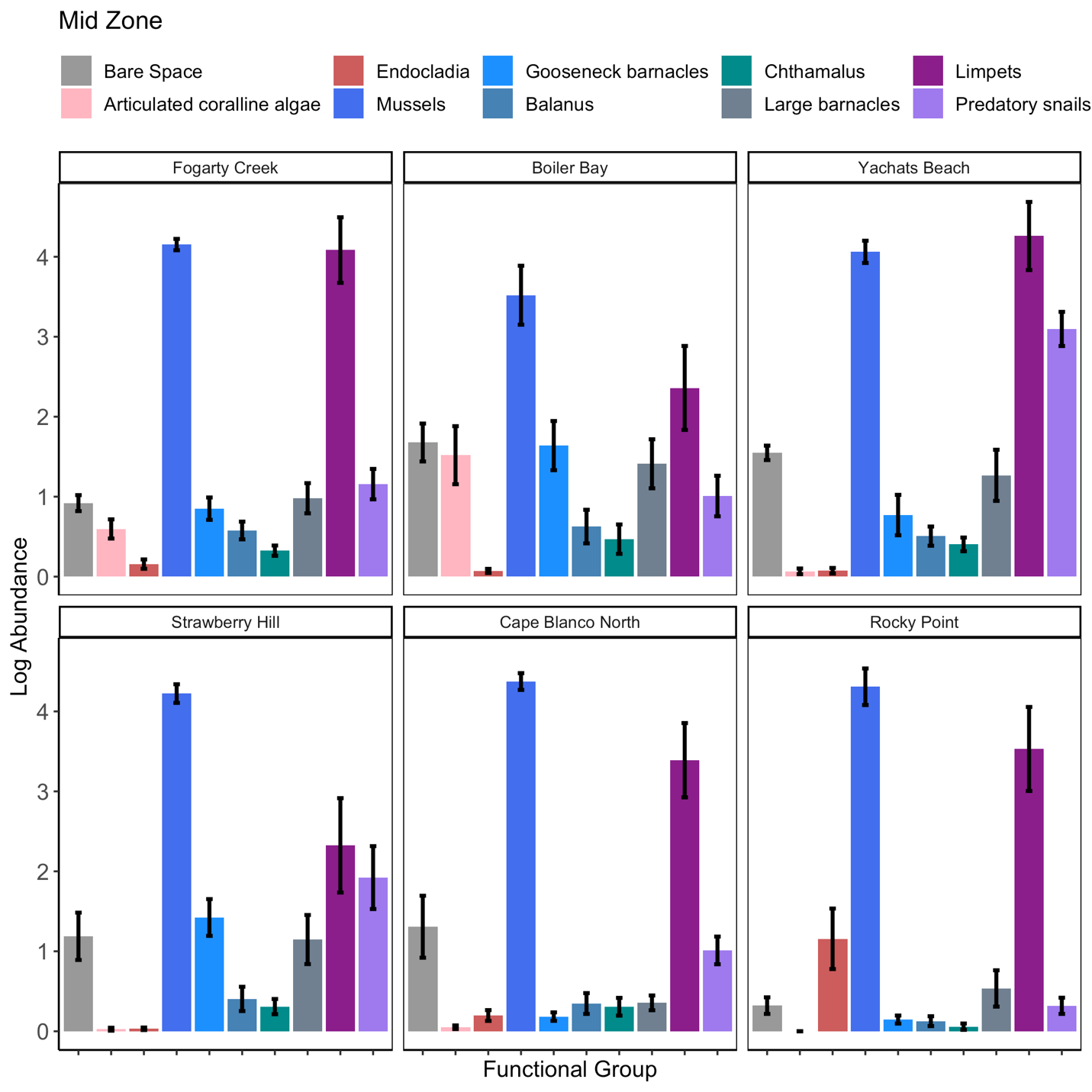


# **Figure S2.** Average (± SE) functional group abundances in the mid intertidal zone among sites in Oregon and northern California from 2006-2020. Colors correspond to functional groups, with bare space in gray, red algae in reds and pinks, sessile invertebrates in blues, and mobile invertebrates in purples.


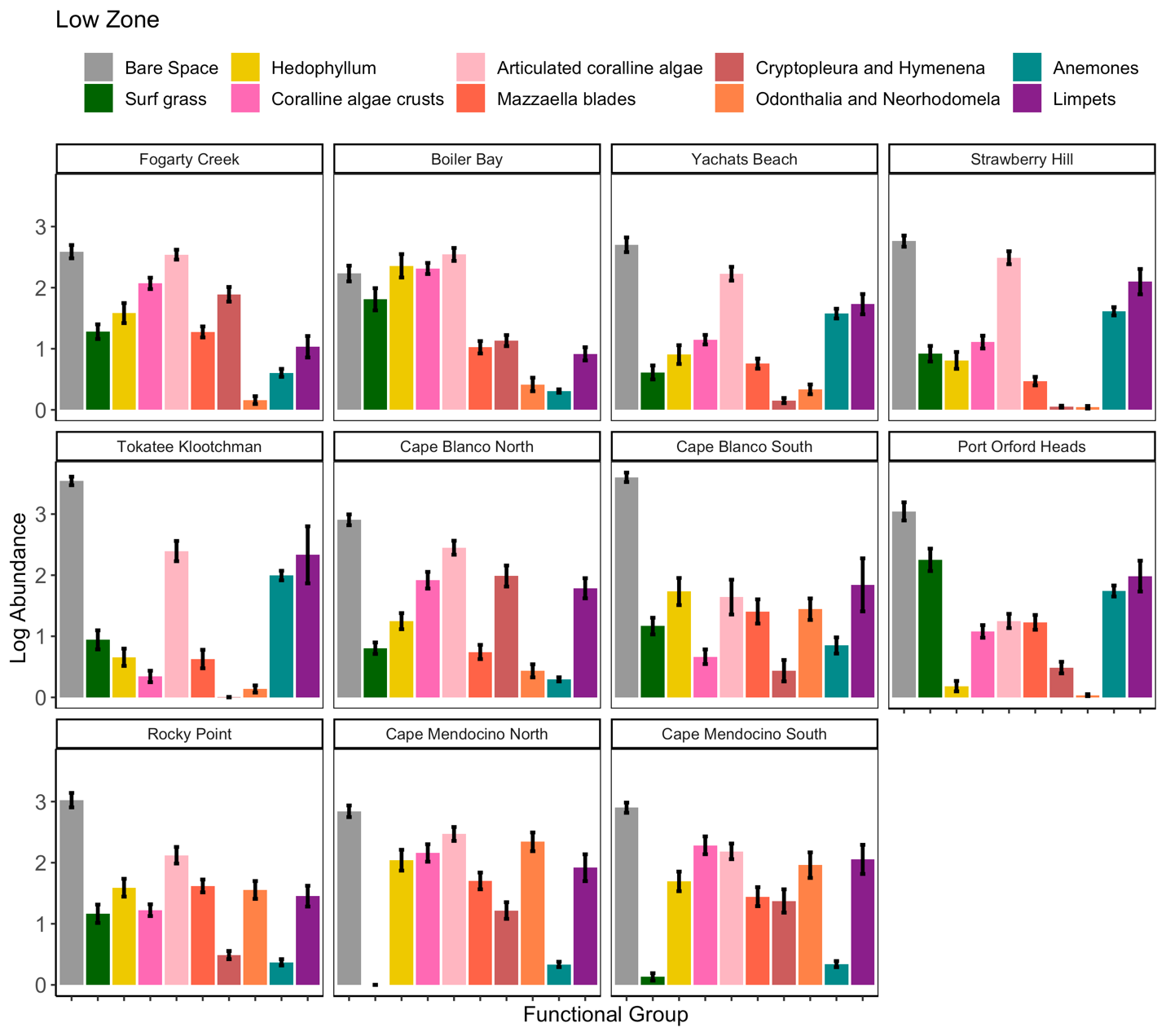


# **Figure S3.** Average (± SE) functional group abundances in the low intertidal zone among sites in Oregon and northern California from 2006-2020. Colors correspond to functional groups, with bare space in gray, green algae/plants in greens, brown algae in yellows, red algae in reds and pinks, sessile invertebrates in blues, and mobile invertebrates in purples.


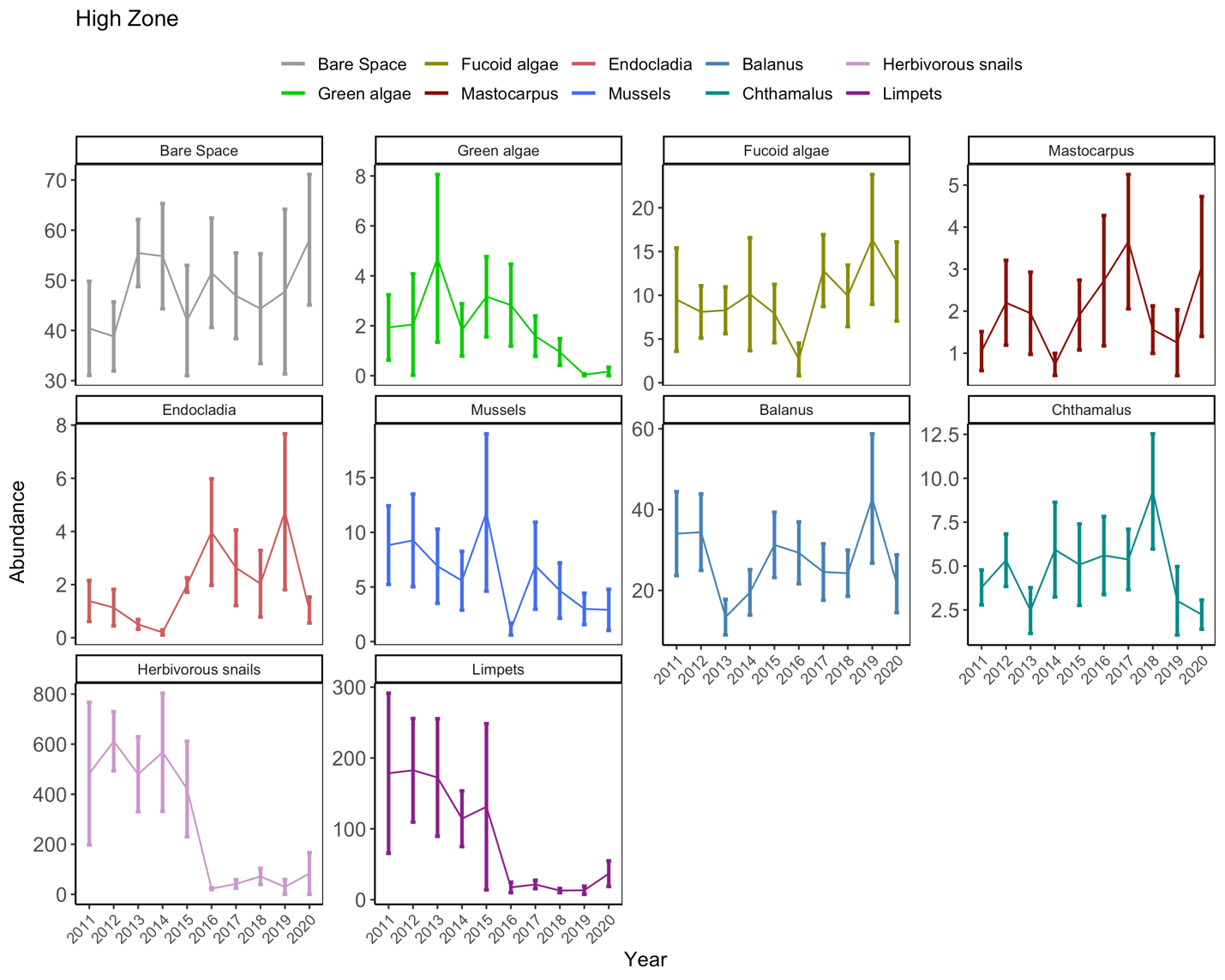


# **Figure S4.** Average (± SE) functional group abundances in the high intertidal zone over time in Oregon and northern California from 2006-2020. Colors correspond to functional groups, with bare space in gray, green algae/plants in greens, brown algae in yellows, red algae in reds and pinks, sessile invertebrates in blues, and mobile invertebrates in purples.


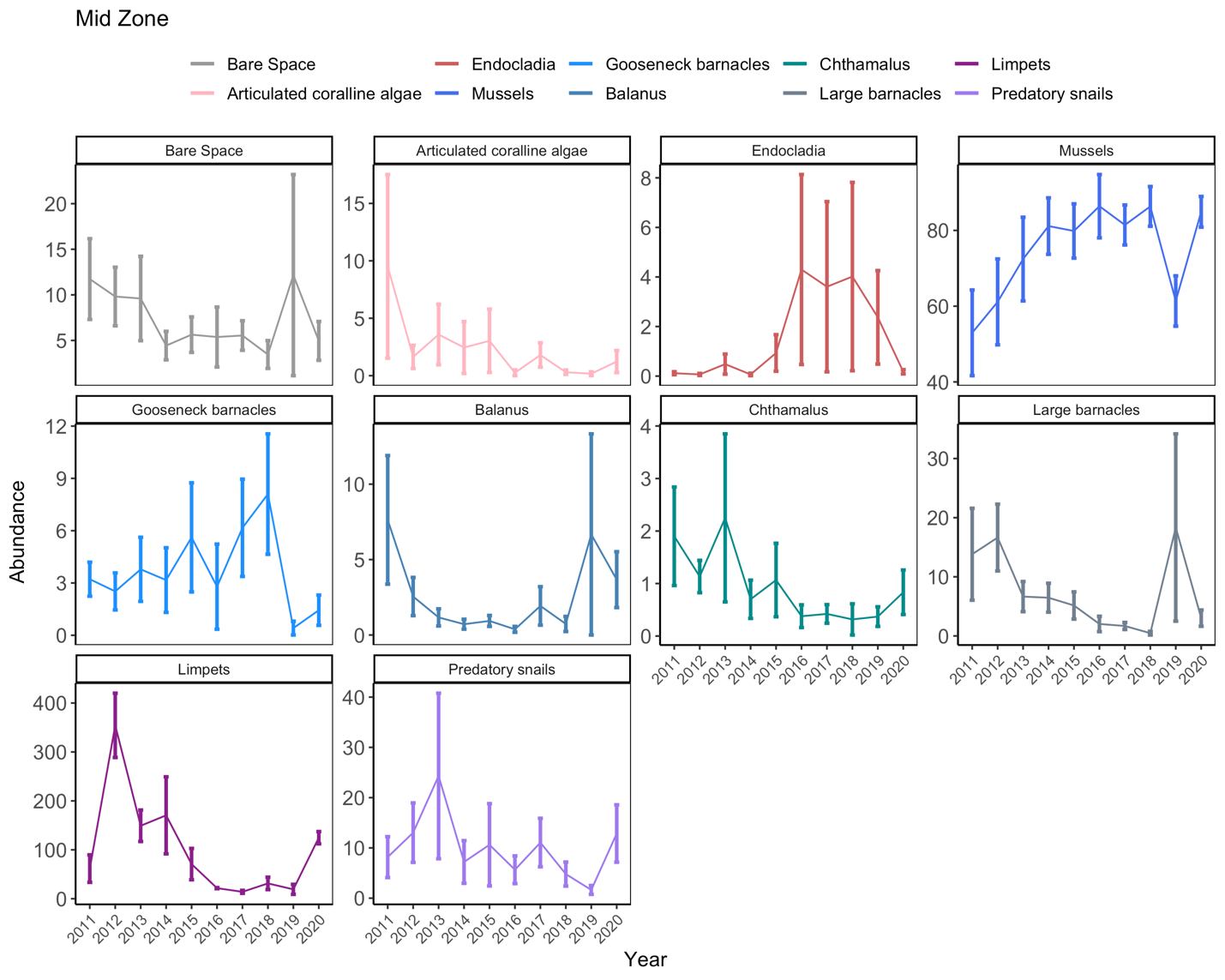


# **Figure S5.** Average (± SE) functional group abundances in the mid intertidal zone over time in Oregon and northern California from 2006-2020. Colors correspond to functional groups, with bare space in gray, red algae in reds and pinks, sessile invertebrates in blues, and mobile invertebrates in purples.


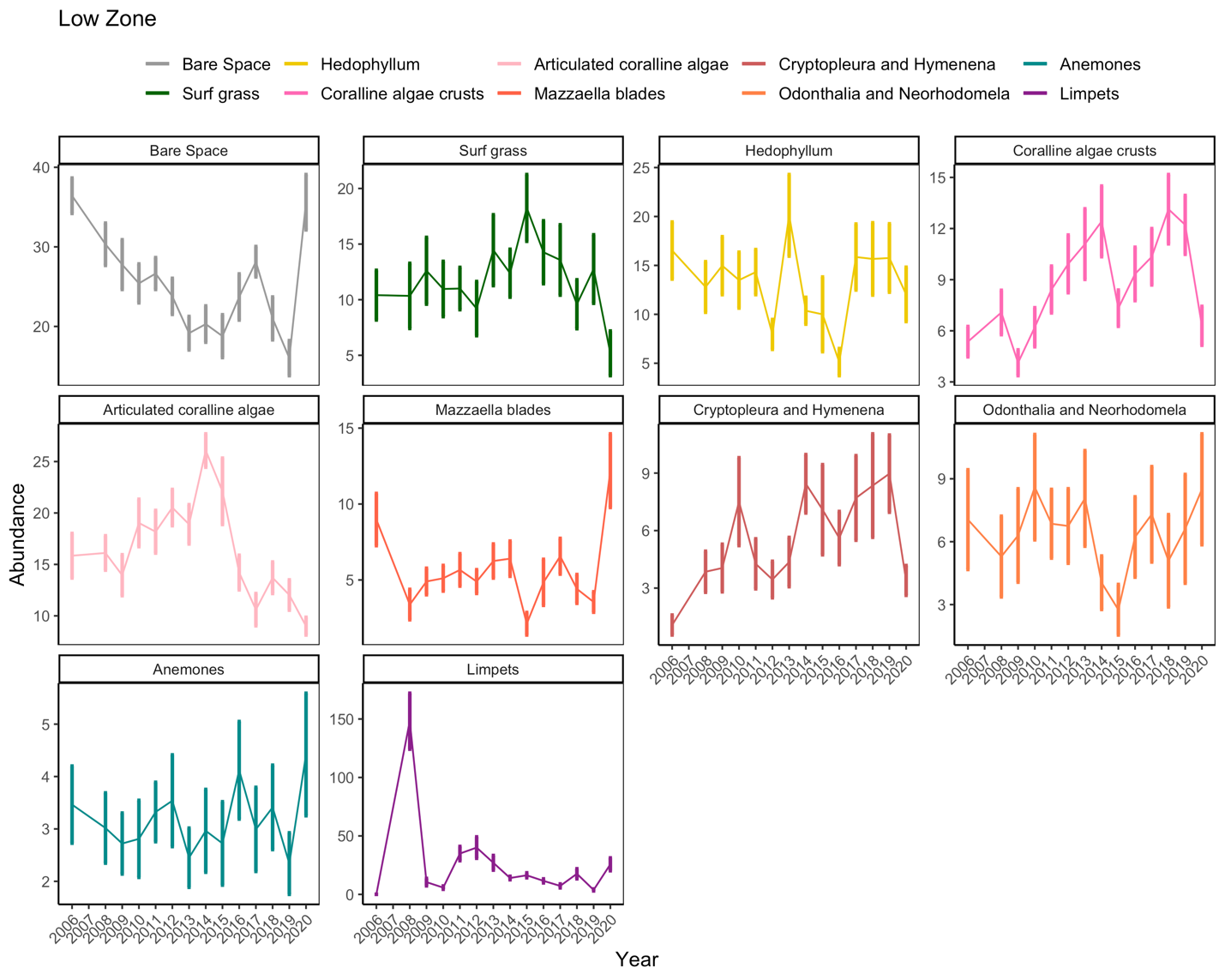


# **Figure S6.** Average (± SE) functional group abundances in the low intertidal zone over time in Oregon and northern California from 2006-2020. Colors correspond to functional groups, with bare space in gray, green algae/plants in greens, brown algae in yellows, red algae in reds and pinks, sessile invertebrates in blues, and mobile invertebrates in purples.


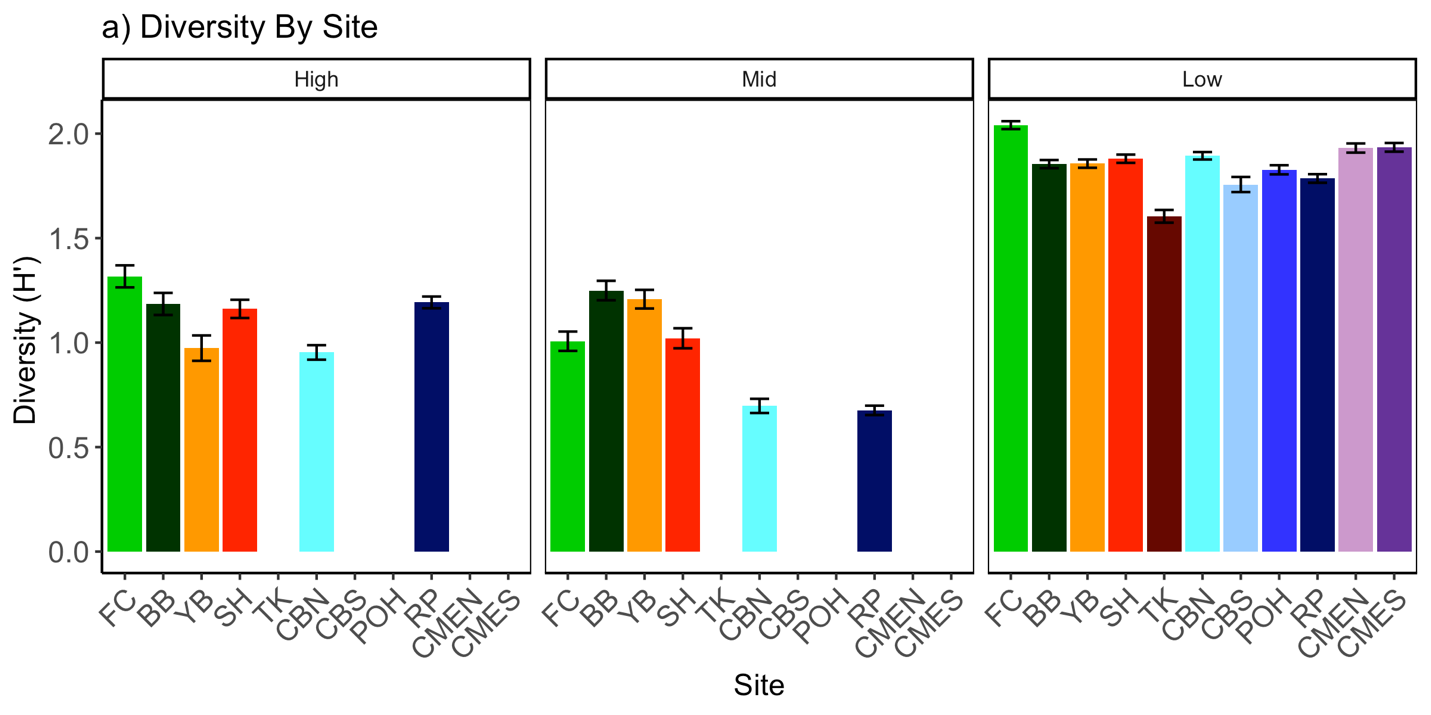

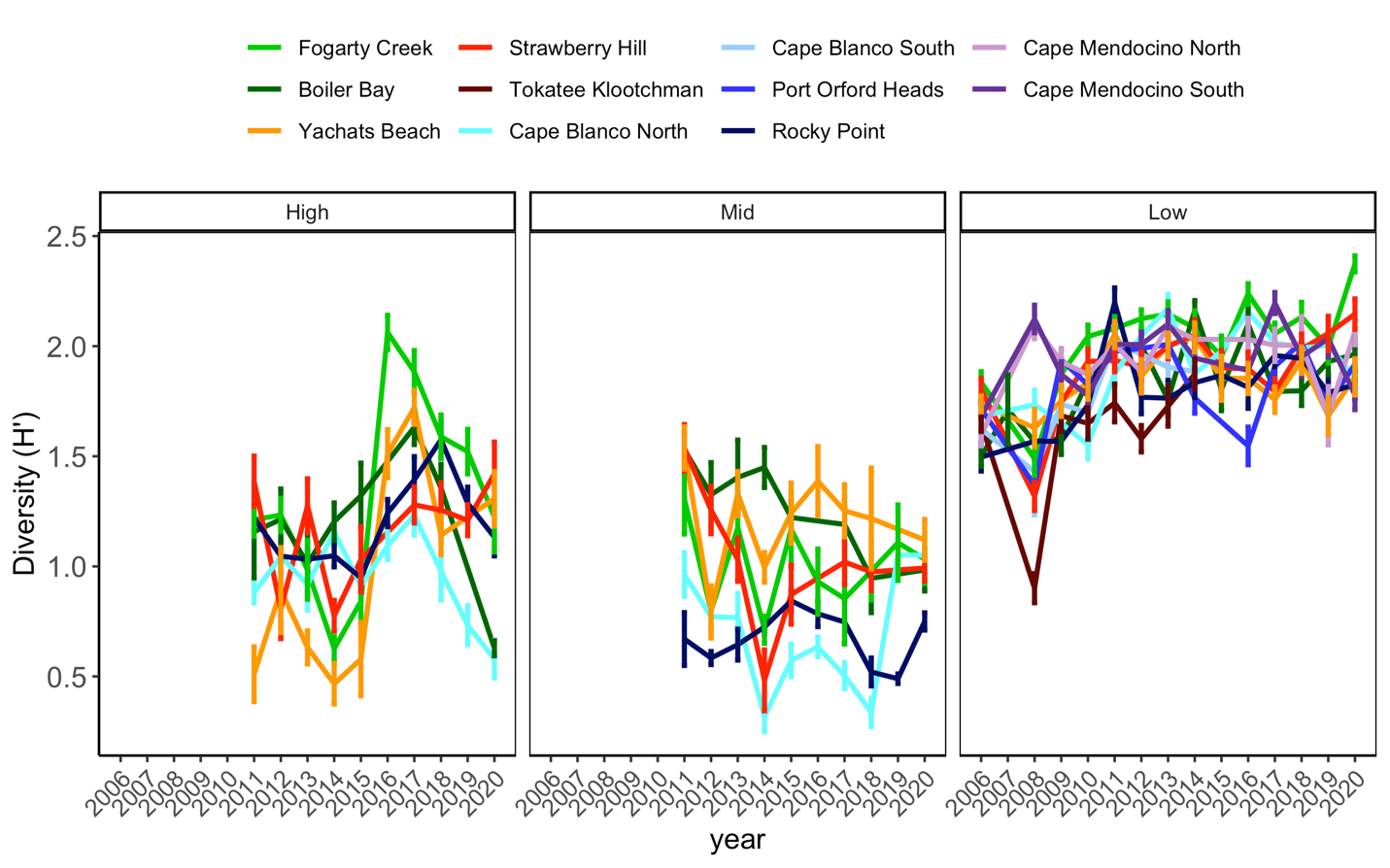


# **Figure S7.** Average (±SE) taxon diversity (Shannon-Weiner index) among a) sites in each zone and b) years in the high (left panels), mid (middle panels) and low (right panels) in Oregon and northern California from 2006-2020. Colors correspond to Cape Foulweather sites in greens, Cape Perpetua sites in reds, Cape Blanco sites in blues, and Cape Mendocino sites in purples.
